# LncRNA LINC00460 promotes EMT in head and neck squamous cell carcinoma by facilitating peroxiredoxin-1 into the nucleus

**DOI:** 10.1186/s13046-019-1364-z

**Published:** 2019-08-20

**Authors:** Yingying Jiang, Wei Cao, Kun Wu, Xing Qin, Xiaoning Wang, Yan Li, Binbin Yu, Zhen Zhang, Xu Wang, Ming Yan, Qin Xu, Jianjun Zhang, Wantao Chen

**Affiliations:** 1 Department of Oral and Maxillofacial-Head & Neck Oncology, Ninth People’s Hospital, Shanghai Jiao Tong University School of Medicine, 639 Zhizaoju Road, Shanghai, 200011 China; 2 Shanghai Key Laboratory of Stomatology & Shanghai Research Institute of Stomatology, National Clinical Research Center of Stomatology, Shanghai, 200011 People’s Republic of China; 3 Department of Dentistry, Affiliated Hospital, Weifang Medical University, Weifang, 261031 China

**Keywords:** LncRNA, LINC00460, PRDX1, EMT, HNSCC

## Abstract

**Background:**

The lncRNA LINC00460 plays crucial roles in several epithelial cancers, although its mechanisms of action differ greatly in different cellular contexts. In this study, we aimed to determine the potential clinical applications of LINC00460 and elucidate the mechanisms by which LINC00460 affects the development and progression of head and neck squamous cell carcinoma (HNSCC).

**Methods:**

The biological functions of LINC00460 were assessed in several epithelial cancer cell lines. The subcellular localization of LINC00460 was evaluated by cell nuclear/cytoplasmic fractionation and fluorescence in situ hybridization. RNA pull-down assays, LS-MS/MS analysis, and RNA and chromatin immunoprecipitation assays were performed to identify the molecular mechanism by which LINC00460 promotes HNSCC progression. The clinical pathological features of LINC00460 and PRDX1 were evaluated in HNSCC tissues and paired adjacent normal tissues.

**Results:**

LINC00460 enhanced HNSCC cell proliferation and metastasis in vitro and in vivo and induced epithelial–mesenchymal transition (EMT). LINC00460 primarily localized within the cytoplasm of HNSCC cells, physically interacted with PRDX1 and facilitated PRDX1 entry into the nucleus. PRDX1 promoted the transcription of LINC00460, forming a positive feedback loop. In addition, PRDX1 also promoted the transcription of EMT-related genes (such as ZEB1, ZEB2 and VIM) through enrichment on gene promoters in the nucleus. LINC00460 effectively induced HNSCC cell EMT in a PRDX1-dependent manner, and PRDX1 mainly mediated the EMT-promoting effect of LINC00460. High levels of LINC00460 and PRDX1 expression were positively associated with lymph metastasis, pathological differentiation and tumor size in HNSCC patients.

**Conclusions:**

LINC00460 promoted EMT in HNSCC cells by facilitating PRDX1 entry into the nucleus. LINC00460 and PRDX1 are promising candidate prognostic predictors and potential targets for cancer therapy for HNSCC.

**Electronic supplementary material:**

The online version of this article (10.1186/s13046-019-1364-z) contains supplementary material, which is available to authorized users.

## Background

Head and neck squamous cell carcinoma (HNSCC) is the most common malignancy of the head and neck epidermis, with over 600,000 new cases reported per year [1, 2]. HNSCCs are heterogeneous, solid and malignant tumors that are associated with low overall survival rates in patients, primarily due to late diagnoses, low therapeutic response rates, and high rates of recurrence and metastasis [3]. Therefore, elucidating the genetic and epigenetic molecular alterations associated with HNSCC is extremely important to improving the diagnosis, appropriate treatment and prognosis of patients with HNSCC.

Various studies have demonstrated that the occurrence and development of HNSCC is closely related to long noncoding RNAs (lncRNAs) [4]. LncRNAs (> 200 nt in length, no protein-coding functions) act as key regulators by participating in gene regulation at the transcriptional, post-transcriptional and post-translational levels [5, 6], and they affect many biological processes [7]. Previous reports have shown that lncRNAs have complex and wide-ranging functions in the development of HNSCC, including functions associated with cancer growth, recurrence and metastasis [8], because of their irregular and specific expression patterns in HNSCC [9]. Although the relationship between lncRNAs and HNSCC is unclear, some lncRNAs have been reported to be aberrantly expressed and to contribute to the occurrence and development of HNSCC [10–12].

Using orthogonal partial least squares discriminant analysis (OPLS-DA), which integrates RNA-Seq data from *The Cancer Genome Atlas* (TCGA) database and matching clinical information from a large cohort of HNSCC patients, we identified LINC00460 as a prognostic lncRNA signature [13]. Analyses of the expression profiles of lncRNAs in HNSCC cells from the Cancer RNA-Seq Nexus (CRN) database have shown that the expression of LINC00460 is upregulated [14, 15]. Located on chromosome 13q33.2 and transcribed as a 913-nt transcript, LINC00460 has been reported to play important roles in tumorigenesis and progression in various tumors and is significantly correlated with survival in the context of several cancer types, including lung cancer [16–19], esophageal cancer [20–22], colorectal cancer [23, 24], nasopharyngeal carcinoma [25], papillary thyroid carcinoma [26], ovarian cancer [27], gastric cancer [28, 29], renal carcinoma [30], meningioma [31], and bladder and urothelial carcinoma [32, 33]*.* According to previous studies, LINC00460 exhibits aberrant expression in and may directly participate in the pathogenesis of HNSCC [13, 34, 35]. The emerging mechanisms of action of LINC00460 differ widely in different cellular contexts; therefore, the key effects and detailed molecular mechanisms of LINC00460 in HNSCC cells remain unclear and urgently need further study and investigation.

To determine whether LINC00460 plays an important role in the occurrence and development of HNSCC and to assess its usefulness as a candidate biomarker for accurate prognostic prediction and as a potential target for cancer therapy, we investigated and identified the functions and mechanisms of action of LINC00460 in HNSCC cells.

## Materials and methods

### Patients and specimens

HNSCC tissues and their paired adjacent normal tissues were obtained from the *Shanghai Sharing Platform for the Tissue and Bioinformatics Database of Oral Maxillofacial Tumors* (http://mdl.shsmu.edu.cn/OMNDB/page/home/home_en.jsp), which was established by the Ninth People’s Hospital, Shanghai Jiao Tong University School of Medicine, and the Shanghai Institute of Stomatology (Shanghai, China). All tissue samples used for the Sharing Platform were collected from the Department of Oral and Maxillofacial-Head and Neck Oncology, Ninth People’s Hospital, Shanghai Jiao Tong University School of Medicine.

### Cell lines and cell culture

Seven human HNSCC cell lines (WSU-HN4, WSU-HN6, WSU-HN30, SCC-4, SCC-9, SCC-25 and CAL-27), a lung cancer cell line (A549) and a cervical cancer cell line (HeLa) were used in this study, and the Research Resource Identifiers (RRIDs) are listed in Additional file 1: Table S1. The WSU-HN4 (HN4), WSU-HN6 (HN6), and WSU-HN30 (HN30) cells were kindly provided by the University of Maryland Dental School, USA, and the A549 and HeLa cells were purchased from the Cell Bank of the Chinese Academy of Sciences, Shanghai, China. These cells were cultured in Dulbecco’s modified Eagle’s medium (DMEM; Gibco-BRL, Grand Island, NY), as were the CAL-27 cells (purchased from the American Type Culture Collection, Manassas, VA). The SCC-4, SCC-9 and SCC-25 cells (also from the American Type Culture Collection) were cultured in DMEM/F12 (1:1) medium (Gibco-BRL). The media were supplemented with 10% heat-inactivated fetal bovine serum (FBS) (Gibco-BRL), penicillin (100 units/mL), and streptomycin (100 μg/mL). The cells were cultured at 37 °C in a humidified 5% CO_2_ atmosphere. In addition, normal oral epithelial cells were primary cultured in keratinocyte serum-free medium (KSF; Gibco-BRL) with 0.2 ng/mL recombinant epidermal growth factor (rEGF; Invitrogen, Carlsbad, CA, USA).

### RNA extraction and qRT-PCR

Total RNA was extracted using TRIzol reagent (TaKaRa, Japan) and used to generate cDNA with a PrimeScript RT Reagent Kit (TaKaRa). All qRT-PCR was performed using an ABI StepOne Real-Time PCR System (Life Technologies, USA) with a TB Green Premix Ex Taq reagent kit (TaKaRa) as previously described [36]. The PCR primers were designed and synthesized by Sangon Biotech (Shanghai) Co., Ltd., and are listed in Additional file 2: Table S2.

### Western blot analysis

Western blotting was performed as previously described [37]. In addition, cytoplasmic and nuclear extracts were separated and prepared using NE-PER™ Nuclear and Cytoplasmic Extraction Reagents (Thermo Fisher Scientific, USA) according to the manufacturer’s instructions and a previous study [36]. Primary antibodies (anti-E-cadherin [Cat# ab15148, 1:500], anti-N-cadherin [Cat# ab18203, 1:1000], anti-ZEB1 [Cat# ab124512, 1:1000], anti-ZEB2 [Cat# ab138222, 1:1000], anti-peroxiredoxin 1/PAG [Cat# ab109498, 1:10,000] and anti-GAPDH [Cat# ab181602, 1:1000] [Abcam, USA]; anti-Vimentin [Cat# D21H3, 1:1000, CST, USA]; anti-H3 [Cat# AH433, 1:1000, Beyotime, China]; and anti-HA Tag [Cat# 26183, 1:10000, Thermo Fisher Scientific]) were used, as well as anti-mouse and anti-rabbit secondary antibodies (IR Dye-labeled secondary antibodies [1:10000, Sigma, USA] and HRP-labeled secondary antibodies [1:10000, CST]). The signals were visualized using an Odyssey Infrared Imaging System (LI-COR Biosciences, USA) or with ECLUltra (New Cell and Molecular Biotech, Suzhou, China).

### Smart Silencer/siRNA or plasmid transfection

The Smart Silencer and siRNA used in our study were designed and synthesized by Guangzhou RiboBio Co., Ltd. (Guangzhou, China), and the sequences were listed in Additional file 3: Table S3. The plasmids were constructed by HanYin Biotechnology Co., Ltd. (Shanghai, China). Transfection was performed using Lipofectamine 3000 reagent (Invitrogen) following the manufacturer’s instructions.

### Lentiviral transduction and screening of stable strains

LINC00460 and PRDX1 lentiviral expression vectors (wild-type and mutant) were constructed by HanYin Biotechnology Co., Ltd. The LINC00460 lentiviral expression vector (LINC00460 vector) conferred puromycin resistance, while the PRDX1 lentiviral expression vector (PRDX1 vector) was C-terminally tagged with an HA epitope and conferred blasticidin resistance. Lentiviral transduction was performed following the manufacturer’s instructions. After 72 h of transfection, the culture medium was mixed with puromycin/blasticidin at a final concentration of 3–10 μg/mL. After being cultured with puromycin/blasticidin and passaged 2–3 times, the stably stained cells were screened.

### Transwell migration and invasion assays

Cell migration and invasion assays were performed using 24-well Transwell chambers with 8-μm porosity polycarbonate filters and Transwell insert chambers (Corning, USA) coated or not coated with Matrigel (BD Biosciences, USA). A total of 150 μL of cell suspension in serum-free medium was added into each upper chamber, while 600 μL of DMEM supplemented with 10% FBS was added to the lower chambers as a chemoattractant. After incubating for 24–36 h, the migrated or invaded cells were fixed with 4% paraformaldehyde (Sangon Biotech) for 15 min and stained with 1% crystal violet (Beyotime) for 30 min. After the cells on the upper surface of the filter were removed, at least five randomly selected microscopic fields of fixed cells per filter were imaged using an inverted phase-contrast microscope. The cells were counted, and the average was calculated.

### Cell counting Kit-8 (CCK-8) analysis

Cells transfected for 24 h with Smart Silencer/siRNA or stably lentivirus-transduced cells were seeded into 96-well plates at a density of 1000 cells per well in triplicate. The cells were harvested, and 10 μL of CCK-8 reagent (Dojindo, Kumamoto, Japan) was added to 100 μL of culture medium. The cells were subsequently incubated for 2 h at 37 °C, and the optical density was measured at 450 nm using a microplate reader (SpectraMax i3, Molecular Devices, USA).

### Colony formation assay

Cells transfected for 24 h with Smart Silencer/siRNA or lentivirus-transduced stable cells were seeded into 6-well plates at a density of 1000 cells per well and incubated for 10–14 days to form cell colonies. The colonies were fixed and stained, and those with more than 50 cells were counted under a dissecting microscope.

### Fluorescence in situ hybridization (FISH) assay

Fluorescence-labeled probes for LINC00460, 18S rRNA, and U6 RNA were designed and synthesized, and FISH experiments were performed using a Ribo™ Fluorescent In Situ Hybridization kit (RiboBio). Images were acquired on a TCS SP2 laser-scanning confocal microscope (Leica Microsystems, Germany).

### Isolation of nuclear and cytoplasmic RNA

Nuclear, cytoplasmic and total RNA was isolated using a PARIS™ kit (Thermo Fisher Scientific) following the manufacturer’s instructions. After purification and DNase I treatment, RNA from the isolated nuclear and cytoplasmic fractions was reverse transcribed and used for PCR as described above. MALAT1, NEAT1, TUG1 and U6 were used as endogenous controls for the nucleus, while BIRC5 and GAPDH were used as endogenous controls for the cytoplasm. The primers used for PCR are listed in Additional file 2: Table S2.

### Immunofluorescence

Cells were seeded onto cover slips in 24-well plates for 24 h, fixed with 4% paraformaldehyde for 20 min and permeabilized with 0.1% Triton X-100 for 10 min. After being blocked in 3% BSA for 30 min, the cells were incubated with E-cadherin (Cat# ab15148, 1:100) or Vimentin antibodies (Cat# D21H3, 1:100) overnight at 4 °C, washed with PBST and then incubated with an Alexa Fluor 549-conjugated anti-goat IgG F (ab’)2 fragment (1:200, Invitrogen) for 1 h at room temperature in the dark. The cells were costained with DAPI (Beyotime) for 5 min for detection of nuclei and then observed and photographed under a Leica TCS-SP2 laser-scanning confocal microscope.

### RNA pull-down assay and liquid chromatography tandem mass spectrometry (LC-MS/MS)

A biotinylated RNA pull-down assay was conducted using a Target RNA Purification kit (ZEHENG Biotech, Shanghai, China) following the manufacturer’s instructions, as previously described [38]. Briefly, CAL-27 cells stably transduced with LINC00460 vector were crosslinked in 1% formaldehyde for 10 min, equilibrated in glycine buffer for 5 min, washed with cold PBS three times, scraped with 1 mL of lysis buffer and incubated for 10 min. The cell samples were sonicated and then centrifuged, after which the supernatant was transferred to a 2-mL tube, and 50 μL was saved for input analysis. The lysate supernatant was incubated with LINC00460 probes (RiboBio) or a negative probe for 3 h at room temperature with rotation; then, 100 μL of Streptavidin Magnetic Beads was added, and the mixture was incubated for 1 h with stirring. The bead/sample mixture was washed twice, after which 10% of the mixture was subjected to RNA purification, while the remaining 90% was subjected to protein purification. After subsequent washes, the pulled-down complexes were analyzed by LC-MS/MS, which was performed by Applied Protein Technology (Shanghai, China). Subsequently, the protein was verified by Western blot analysis after RNA pull-down assays were performed.

RNA purification was performed as described previously [38], and the efficiency of target purification was assessed by qRT-PCR.

### RNA immunoprecipitation (RIP)

RIP was performed using an EZ-Magna RIP™ RNA-Binding Protein Immunoprecipitation Kit (Millipore, Billerica, MA, USA) according to the manufacturer’s instructions. After cell lysis with RIP lysis buffer, 100 μL of the lysate was incubated with RIP buffer containing magnetic beads, which were conjugated with human anti-HA (Thermo Fisher Scientific) and normal rabbit IgG (Millipore). Among the antibodies, IgG was considered a negative control (NC). Proteinase K buffer was then added to the samples. Finally, the target RNA was extracted and purified for further study by qRT-PCR assays. The primers used for PCR are listed in Additional file 4: Table S4.

### Chromatin immunoprecipitation (ChIP)

ChIP assays were performed on CAL-27 cells stably transduced with PRDX1-HA vector with a ChIP assay kit (Millipore) according to the manufacturer’s instructions. IgG was used as the NC, and an anti-HA antibody (Thermo Fisher Scientific) was used to pull down the promoter regions of LINC00460, ZEB1, ZEB2 and VIM genes with the PRDX1 regulatory element. The DNA fragments were purified with a DNA Clean kit (Beyotime) and used for qPCR with primers for the promoters of LINC00460, ZEB1, ZEB2 and VIM (Additional file 5: Table S5). The results are presented as fold changes and were calculated by dividing the signals from ChIP obtained with the anti-HA antibody by those obtained with the IgG control.

### Xenograft formation and in vivo metastasis assay

All animal experiments, implemented in BALB/C nude mice (4 weeks old) (Shanghai Laboratory Animal Center, Shanghai, China), were conducted in accordance with the appropriate ethical standards and national guidelines.

To assess whether LINC00460 knockdown could inhibit tumorigenic capacity in vivo, we established a xenograft tumor mouse model using cholesterol-conjugated LINC00460 siRNA (si-LINC00460) for in vivo siRNA delivery. A total of 1 × 10^6^ CAL-27 cells in 100 μL of serum-free DMEM were subcutaneously injected into the left and right dorsal flanks of six mice. Ten days after tumor inoculation, cholesterol-conjugated si-LINC00460 (sequences shown in Additional file 3: Table S3) from RiboBio were used for in vivo siRNA delivery in three mice, while another three mice were injected with NC siRNA. When the size of the tumors reached approximately 5 mm × 5 mm, siRNA (10 nmol in 0.1 mL of saline buffer per tumor nodule) was injected into the tumor mass once every 3 days for 3 weeks according to the methods in a previous study [39].

To determine whether LINC00460 overexpression could enhance tumorigenicity in vivo, 1 × 10^6^ CAL-27 cells stably transduced with LINC00460 vector or NC cells were subcutaneously injected into the right and left flanks of six mice, respectively.

During the xenograft tumor experiments, the tumor sizes were monitored with a caliper every 3 days. The tumor volume was measured using the following formula: tumor volume = length×width×width/2. After the animals were sacrificed, the tumor samples were collected, and the weights were measured. The samples were embedded in paraffin for further hematoxylin and eosin (H&E) staining and immunohistochemistry (IHC) analysis.

Due to the weak invasive characteristics of HNSCC cell lines, we used A549 cells for the animal metastasis assay to verify the in vivo functions of LINC00460 via mouse tail vein injection. In our study, we administered tail vein injections with 2 × 10^6^ A549 cells stably transduced with LINC00460 vector or NC cells into two groups of eight mice each. After 8 weeks, the mice were sacrificed, and the lungs were collected. The metastatic nodules formed on the lung surfaces were examined by picric acid and neutral aldehyde staining and further H&E staining and IHC analysis.

### IHC

Excised tumor and lung tissues were fixed in 4% paraformaldehyde, dehydrated, paraffin-embedded, and cut into sections. Consecutive 4-μm-thick sections were analyzed using primary antibodies against Ki-67 (Cat# ab833, 1:50, Abcam) and a biotin-conjugated goat anti-rabbit polyclonal antibody (1:50; ZSGB-BIO, Beijing, China) as the secondary antibody. Images were obtained by light microscopy (Olympus, Japan) at 100× and 400× magnification and quantified using Image-Pro Plus. Five random fields were examined per animal.

### Statistical analysis

All statistical analyses were performed using Statistical Package for Social Science software Version 16.0 (SPSS 16.0) and GraphPad Prism 7.0. The data are presented as the mean ± standard deviation (SD) and are representative of at least three independent experiments. Differences among groups were analyzed by one-way analysis of variance (ANOVA) or *t*-tests (two groups). Analyses of associations between LINC00460 or PRDX1 mRNA levels and clinical features were performed using the Mann-Whitney U-test. The correlation between LINC00460 and PRDX1 was determined by Pearson analysis. All *p* values< 0.05 were considered to indicate statistical significance.

## Results

### LINC00460 facilitates HNSCC cell proliferation, migration and invasion in vitro

**Fig. 1:**
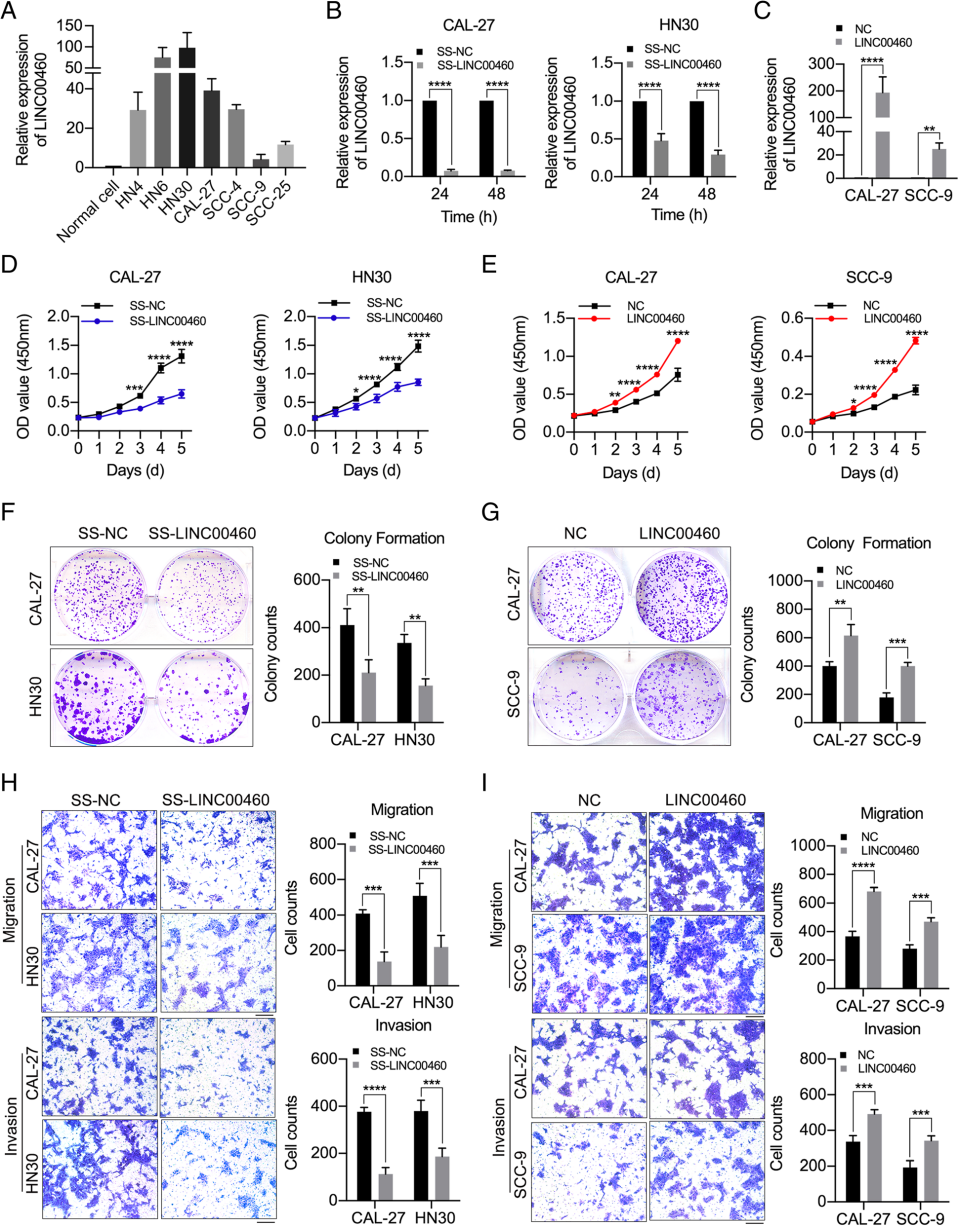
LINC00460 facilitated HNSCC cell proliferation, migration and invasion in vitro. **a** The relative expression of LINC00460 in 7 HNSCC cell lines and normal oral epithelial cells (Normal cell) was detected by qRT-PCR. **b** The relative expression of LINC00460 in CAL-27 and HN30 cells transfected with SS-LINC00460 was detected at 24 and 48 h by qRT-PCR. **c** The relative expression of LINC00460 in CAL-27 and SCC-9 cells stably transduced with LINC00460 vector (LINC00460) was detected by qRT-PCR. **d**, **e** The effect of LINC00460 on cell proliferation was evaluated with CAL-27 and HN30 cells transfected with SS-LINC00460 (**d**) and with CAL-27 and SCC-9 cells stably transduced with LINC00460 (**e**) by CCK-8 assays. **f**, **g** The colonizing ability of CAL-27 and HN30 cells transfected with SS-LINC00460 (**f**) and of CAL-27 and SCC-9 cells stably transduced with LINC00460 (**g**) was determined by colony formation assays. **h**, **i** The cell migration and invasion abilities of CAL-27 and HN30 cells transfected with SS-LINC00460 (**h**) and of CAL-27 and SCC-9 cells stably transduced with LINC00460 (**i**) were determined by transwell assays, Scale bar: 1000 μm. (NC: negative control; SS-LINC00460: LINC00460 Smart Silencer; LINC00460 vector: LINC00460 lentiviral expression vector.) *p < 0.05, **p < 0.01, ***p < 0.001, ****p < 0.0001

LINC00460 was upregulated in 7 HNSCC cell lines compared with normal oral epithelial cells (*p* < 0.05). The expression of LINC00460 was highest in HN30 cells and lowest in SCC-9 cells, with CAL-27 exhibiting medium expression (Fig. 1a). Therefore, we performed loss- and gain-of-function with HN30, SCC-9 and CAL-27 cell lines to elucidate the biological functions of LINC00460 in HNSCC cells. CAL-27 and HN30 cells were transfected with a Smart Silencer specifically targeting LINC00460 (SS-LINC00460), while CAL-27 and SCC-9 cells were stably transduced with LINC00460 vector (Additional file 6: Figure S1A).

The LINC00460 Smart Silencer (SS-LINC00460) effectively knocked down LINC00460 expression in CAL-27 and HN30 cells (Fig. 1b). In contrast, LINC00460 expression was dramatically increased in CAL-27 and SCC-9 cells transduced with LINC00460 vector (Fig. 1c). The CCK-8 assay and colony formation assay results showed that LINC00460 knockdown suppressed cell proliferation in CAL-27 and HN30 cells (Fig. 1d and f), whereas LINC00460 overexpression enhanced cell proliferation in CAL-27 and SCC-9 cells (Fig. 1e and g). The results of transwell assays demonstrated that LINC00460 knockdown suppressed cell migration and invasion in CAL-27 and HN30 cells (Fig. 1h), while LINC00460 overexpression enhanced cell migration and invasion in CAL-27 and SCC-9 cells (Fig. 1i).

In addition, A549 and HeLa cells were used for functional verification of LINC00460 to determine whether LINC00460 showed the similar oncogenic functions in these cell lines (Additional file 6: Figure S1B). The results showed that LINC00460 could enhance cell proliferation and migration in A549 and HeLa cells, as demonstrated by the CCK-8 assay (Additional file 6: Figure S1C), colony formation assay (Additional file 6: Figure S1D) and transwell assay results (Additional file 6: Figure S1E). The results obtained using A549 and HeLa cells were consistent with those obtained using HNSCC cells.

### LINC00460 induced the EMT phenotype and was primarily localized in the cytoplasm

**Fig. 2:**
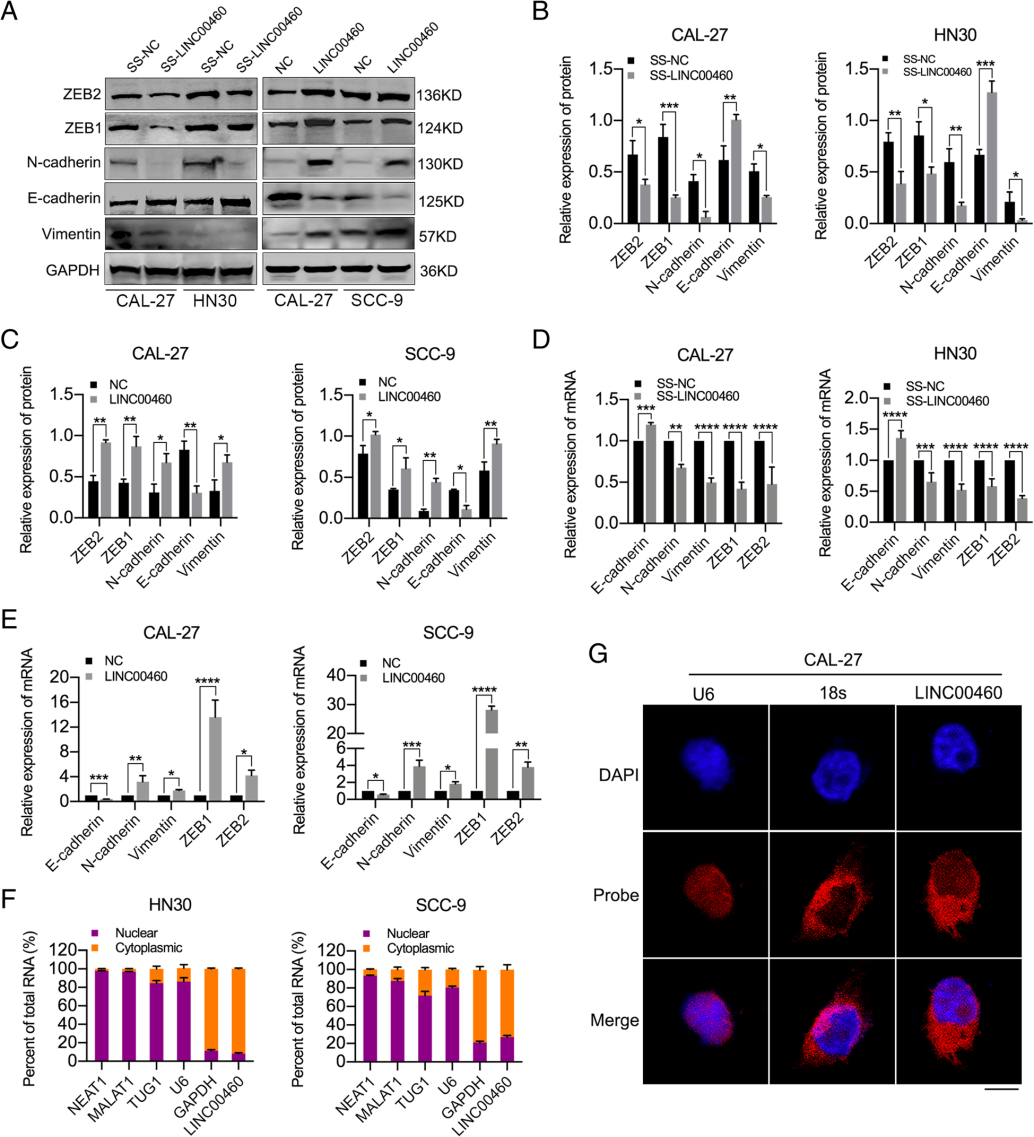
LINC00460 induced the EMT phenotype and was primarily localized in the cytoplasm. **a**-**c** The expression of some EMT markers (E-cadherin, N-cadherin, Vimentin, ZEB1 and ZEB2) was detected by Western blot analysis when LINC00460 was knocked down or overexpressed in HNSCC cells. **d**, **e** The expression of E-cadherin, N-cadherin, Vimentin, ZEB1 and ZEB2 was detected by qRT-PCR when LINC00460 was knocked down (**d**) or overexpressed (**e**) in HNSCC cells. **f** Cell nuclear/cytoplasmic fractionation and qRT-PCR showed the cellular distribution of LINC00460 in HN30 and SCC-9 cells. (NEAT1, TUG1, MALAT1, BIRC5, U6 and GAPDH were used as separation quality standards and endogenous controls). **g** FISH analysis of LINC00460 in CAL-27 cells. (The nuclei were stained with DAPI, and 18S rRNA was used as a cytoplasmic marker. Scale bar: 10 μm.) *p < 0.05, **p < 0.01, ***p < 0.001, ****p < 0.0001

LINC00460 knockdown significantly increased the levels of E-cadherin but decreased those of N-cadherin, Vimentin, ZEB1 and ZEB2 in CAL-27 and HN30 cells, whereas LINC00460 overexpression significantly decreased the levels of E-cadherin but increased those of N-cadherin, Vimentin, ZEB1 and ZEB2 in CAL-27 and SCC-9 cells, as determined by Western blot (Fig. 2a-c) and qRT-PCR analyses (Fig. 2d and e). Moreover, LINC00460 overexpression in CAL-27 cells was accompanied by decreases in E-cadherin and increases in Vimentin levels, as observed in immunofluorescence assays (Additional file 7: Figure S2A). Consistent with the results in HNSCC cells, LINC00460 overexpression significantly decreased the levels of E-cadherin but increased those of N-cadherin and Vimentin in A549 and HeLa cells, as determined by Western blot assays (Additional file 7: Figure S2B).

To determine the subcellular localization of LINC00460 in HNSCC cells, we performed cytoplasmic/nuclear fractionation with HN30 and SCC-9 cells and FISH assays with CAL-27 cells. The amount of LINC00460 in the cytoplasm was higher than that observed in the nucleus, revealing that LINC00460 is predominantly located in the cytoplasm (Fig. 2f and g).

### LINC00460 promoted HNSCC cell growth and metastasis in vivo

**Fig. 3:**
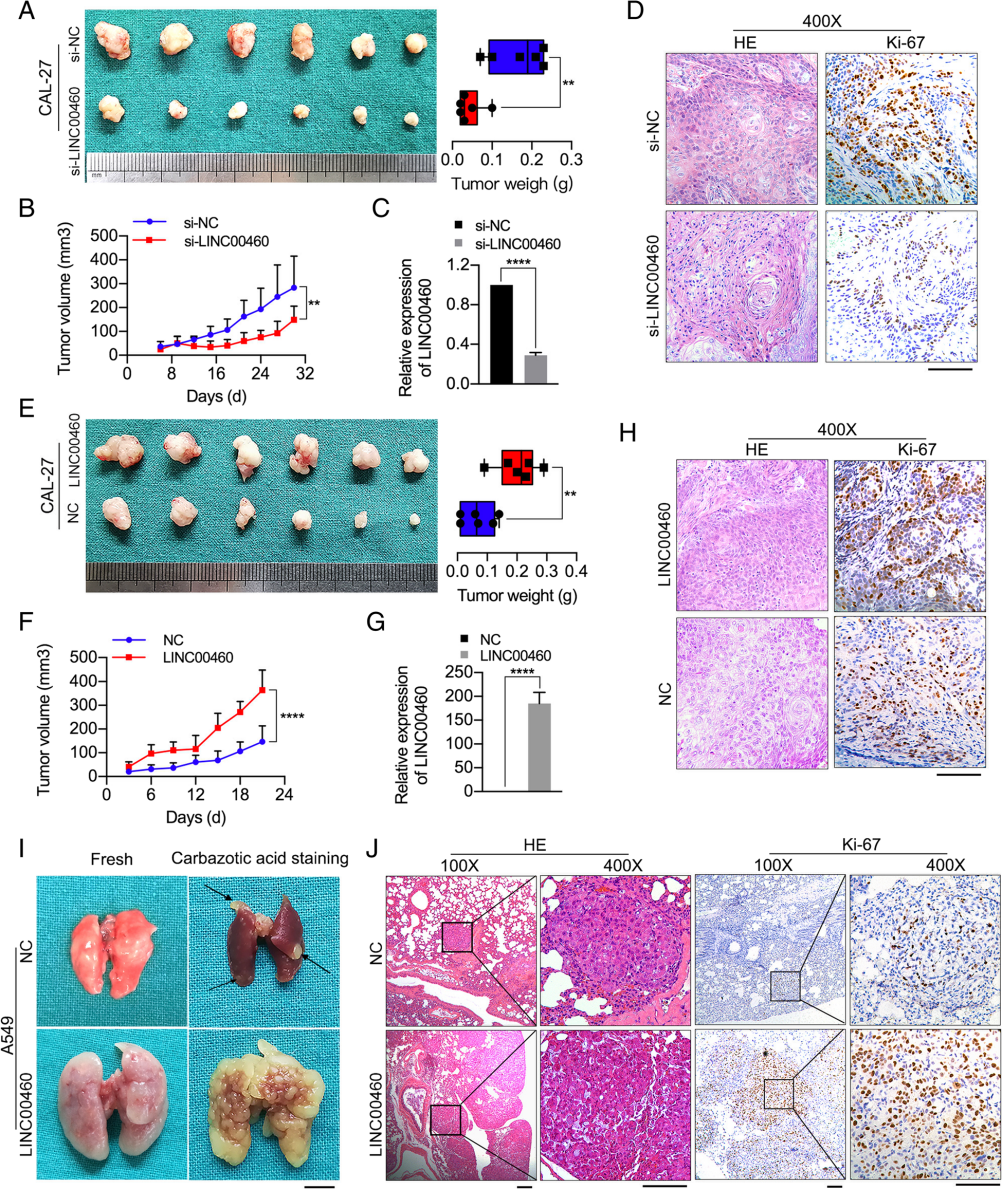
LINC00460 promoted HNSCC cell growth and metastasis in vivo. **a** The volumes and weights of tumors from CAL-27 tumor-bearing nude mice treated with cholesterol-conjugated si-LINC00460 or si-NC are shown *n* = 6/group, **p < 0.01. **b** The tumor growth curves of tumors from CAL-27 tumor-bearing nude mice treated with cholesterol-conjugated si-LINC00460 or si-NC are shown. **p < 0.01. **c** The levels of LINC00460 expression in tumor tissues formed from CAL-27 cells treated with si-LINC00460 and si-NC as determined by qRT-PCR. ****p < 0.0001. **d** H&E staining and IHC of Ki-67 in tumor tissues. Scale bar: 100 μm. **e** The volumes and weights of tumors in nude mice subcutaneously inoculated with CAL-27 cells stably transduced with LINC00460 at the end of the experiment are shown. *n* = 6/group, **p < 0.01. **f** The tumor volumes were calculated every 3 days for 3 weeks, and the tumor growth curves are shown. ****p < 0.0001. **g** The expression of LINC00460 in tumor tissues formed from CAL-27 cells stably transduced with LINC00460 was detected by qRT-PCR. ****p < 0.0001. **h** H&E staining and IHC of Ki-67 expression in tumor tissue. Scale bar: 100 μm. **i** Representative images of the lungs of mice inoculated with A549 cells stably transduced with LINC00460 or NC by tail vein injection for 8 weeks. The arrows show yellow nodules on the lung surfaces. Scale bar: 5 mm. **j** H&E staining and IHC of Ki-67 expression in lung tissues with tumor colonization. Scale bar: 50 μm

To identify the siRNA specifically targeting LINC00460 for animal experiments, three siRNAs contained in SS-LINC00460 were synthetized, and the silencing efficiency was detected by qRT-PCR. The results showed that si-LINC00460–1 was the most effective in knocking down LINC00460 expression (Additional file 8: Figure S3). Therefore, si-LINC00460–1 (si-LINC00460) was used to perform siRNA treatment in vivo and following rescue experiment in vitro. Knockdown of LINC00460 by siRNA treatment significantly decreased tumor growth, as shown by the significantly reduced tumor volumes and weights in the knockdown group compared with the control group (Fig. 3a and b). Furthermore, the expression of LINC00460 in xenograft tumor tissues was confirmed by qRT-PCR, which showed that LINC00460 expression was significantly decreased in si-LINC00460-treated subcutaneous xenografts compared to control groups (Fig. 3c). The results from H&E and IHC staining of Ki-67 further confirmed the alterations in tumor formation (Fig. 3d).

In contrast, significantly larger tumor volumes and weights were observed in mice subcutaneously injected with CAL-27 cells stably transduced with LINC00460 vector (Fig. 3e and f). The overexpression of LINC00460 in xenograft tumor tissues was confirmed by qRT-PCR (Fig. 3g), and the results of H&E and Ki-67 staining further confirmed the alterations in tumor formation (Fig. 3h).

Eight weeks after tail vein injections, the mice were sacrificed, and the metastatic nodules formed on the lung surfaces were examined. As shown in Fig. 3i, the lungs of mice injected with A549 cells stably transduced with LINC00460 vector exhibited significantly increased volumes, whiter color, more and larger solid nodules and more stable textures than the lungs of the control group. The presence of metastatic nodules in the mouse lungs was confirmed by H&E and Ki-67 staining, and the mice injected with A549 cells transduced with LINC00460 vector formed more nodules on their lung surfaces than the control group (Fig. 3j).

### PRDX1 physically interacted with LINC00460 and affected HNSCC cell proliferation and migration

**Fig. 4:**
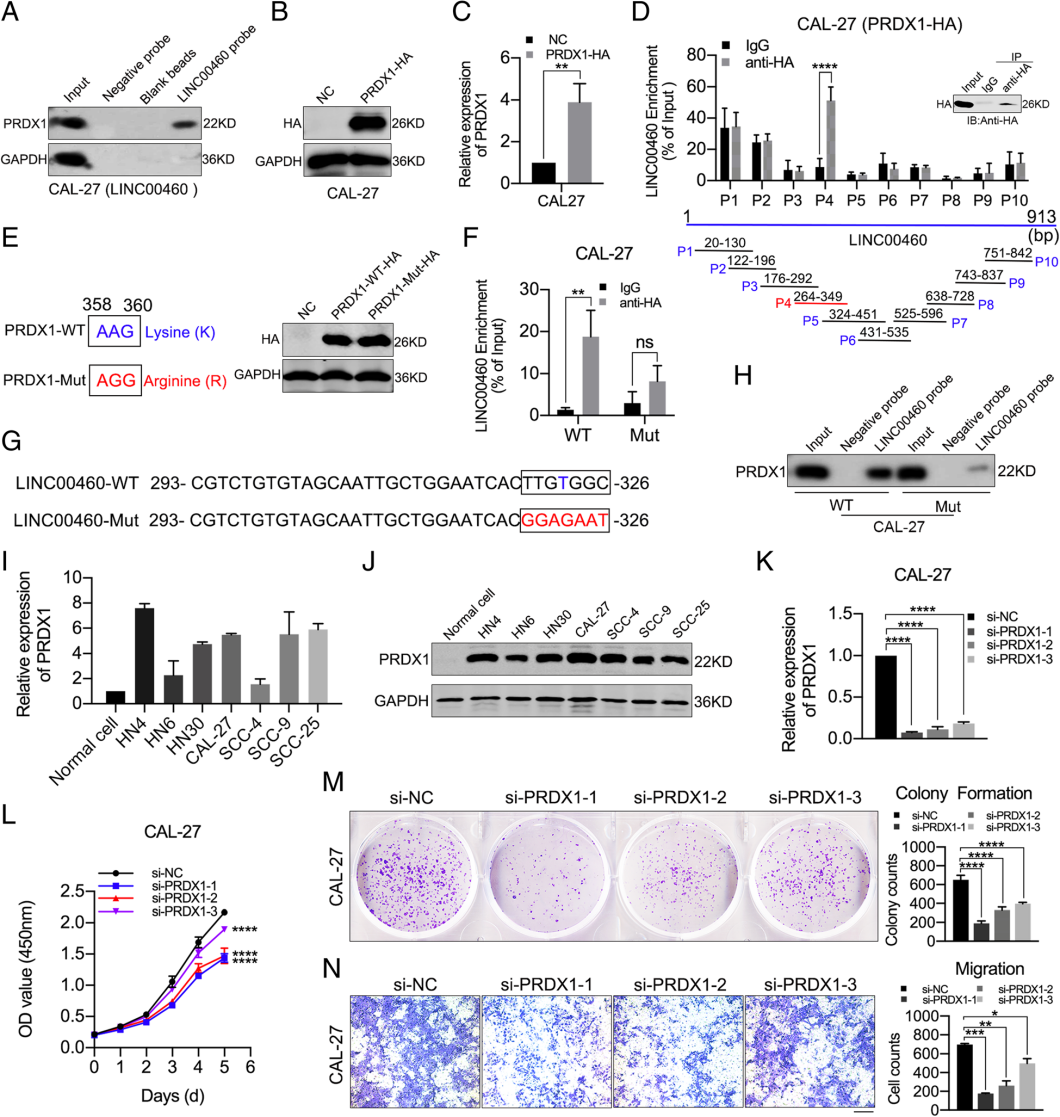
PRDX1 physically interacted with LINC00460 and affected HNSCC cell proliferation and migration. **a** Western blot analysis of PRDX1 following RNA pull-down assays with LINC00460 chromatin isolation by LINC00460 probes in CAL-27 cells stably transduced with LINC00460. **b**, **c** Relative expression of PRDX1 in CAL-27 cells infected with the PRDX1 lentiviral expression vector with HA-Tag (PRDX1-HA) as detected by Western blot analysis (**b**) and qRT-PCR (**c**). **d** qRT-PCR analysis of LINC00460 enriched by anti-HA in CAL-27 cells stably transduced with PRDX1-HA in RIP assays. Ten specific primers for LINC00460 were used to detect the results of RNA enrichment. **e** Construction of the PRDX1-Mut vector, which changed AAG to AGG from nucleotides 358 to 360, and the relative expression of PRDX1-WT and PRDX1-Mut vectors as detected by Western blot analysis in CAL-27 cells. **f** qRT-PCR analysis of LINC00460 enriched by anti-HA in CAL-27 cells transfected with PRDX1-HA-WT (WT) and PRDX1-HA-Mut (Mut) vectors in RIP assays. **g** Construction of the LINC00460-Mut vector, which changed TTGTGGC into GGAGAAT from nucleotides 320 to 326. **h** Western blot of PRDX1 following RNA pull-down assays retrieved by LINC00460 probes in CAL-27 cells transfected with LINC00460-WT (WT) and LINC00460-Mut (Mut) vectors. **i**, **j** The relative expression of PRDX1 in 7 HNSCC cell lines and normal oral epithelial cells (Normal cell) as determined by qRT-PCR (**i**) and Western blot analysis (**j**). **k** Silencing efficiency of si-PRDX1 in CAL-27 cells as detected by qRT-PCR. **l** The effect of PRDX1 expression on cell proliferation was evaluated with CAL-27 cells transfected with si-PRDX1 by CCK-8 assays. **m** The colonizing ability of CAL-27 cells transfected with si-PRDX1 was determined by colony formation assays. **n** The cell migration abilities of CAL-27 cells transfected with si-PRDX1 were determined by transwell assays, Scale bar: 1000 μm. *p < 0.05, **p < 0.01, ***p < 0.001, ****p < 0.0001, ns: no significance

To investigate the mechanism by which LINC00460 affects cell proliferation, migration and EMT, we explored the putative RNA-binding proteins (RBPs) interacting with LINC00460 using RNA pull-down assays (Additional file 9: Figure S4A) followed by mass spectrometry (Additional file 9: Figure S4B and C). Western blot analysis was performed following the RNA pull-down assay to confirm that PRDX1 was an RBP binding with LINC00460 (Fig. 4a). Then, a PRDX1 lentiviral expression vector (PRDX1 vector) with HA-Tag (PRDX1-HA) was constructed (Additional file 9: Figure S4D). CAL-27 and HN30 cells stably transduced with PRDX1-HA exhibited dramatically increased the expression of PRDX1, as determined by Western blot analysis (Fig. 4b and Additional file 9: Figure S4E) and qRT-PCR (Fig. 4c and Additional file 9: Figure S4F). The interaction between PRDX1 and LINC00460 was confirmed in CAL-27 and HN30 cells transduced with PRDX1-HA by RIP assays, and the fourth RIP primer (P4) was shown to produce positive amplification (Fig. 4d and Additional file 9: Figure S4G). The possible binding sites between PRDX1 and LINC00460 were predicted by Protein–RNA Interaction predictor (PRIdictor, http://bclab.inha.ac.kr/pridictor) [40]. A possible binding site on PRDX1 for LINC00460 was located at the lysine (K) residue at amino acid (aa) position 120 (Additional file 9: Figure S4H). A PRDX1 mutant vector (PRDX1-Mut) was constructed based on the predicted LINC00460 binding site (Fig. 4e). After the K at aa 120 was mutated to arginine (R), the ability of PRDX1 to bind LINC00460 was significantly weakened, which suggested that the K at aa 120 of PRDX1 was important for interaction with LINC00460 (Fig. 4f and Additional file 9: Figure S4I). In the PRIdictor database, there were four putative PRDX1-binding sites on LINC00460 (Additional file 9: Figure S4J and K). Based on the results obtained with the fourth RIP primer, we speculate that nucleotide 323 of LINC00460 might be responsible for binding with PRDX1. Therefore, a LINC00460 mutant vector (LINC00460-Mut) was constructed based on the predicted PRDX1-binding site (U at nucleotide 323) (Fig. 4g), and RNA pull-down assays were performed on CAL-27 cells transfected with LINC00460-WT or LINC00460-Mut vectors. Minimal PRDX1 could be pulled down by the LINC00460 probe in CAL-27 cells transfected with the LINC00460-Mut vector, suggesting that the nucleotides at positions 323 of LINC00460 may be responsible for the ability of LINC00460 to bind with PRDX1 (Fig. 4h).

PRDX1 was observed to be upregulated in 7 HNSCC cell lines in qRT-PCR (Fig. 4i) and Western blot assays (Fig. 4j). siRNAs specifically targeting PRDX1 (si-PRDX1) were synthetized, and the silencing efficiency of the si-PRDX1 was detected by qRT-PCR in CAL-27 and HN30 cells (Fig. 4k and Additional file 10: Figure S5A) and by Western blotting (Additional file 10: Figure S5B and C), the results of which showed that si-PRDX1–1 was the most effective in knocking down PRDX1 expression.

The results of the CCK-8 (Fig. 4l and Additional file 10: Figure S5D) and colony formation assays (Fig. 4m and Additional file 10: Figure S5E) showed that PRDX1 knockdown suppressed cell proliferation in CAL-27 and HN30 cells (*p* < 0.05). In addition, the results of the transwell assays demonstrated that PRDX1 knockdown suppressed cell migration in both CAL-27 and HN30 cells (*p* < 0.05) (Fig. 4n and Additional file 10: Figure S5F).

### LINC00460 facilitated PRDX1 entry into the nucleus, and PRDX1 promoted the transcription of LINC00460 and EMT-related genes

**Fig. 5:**
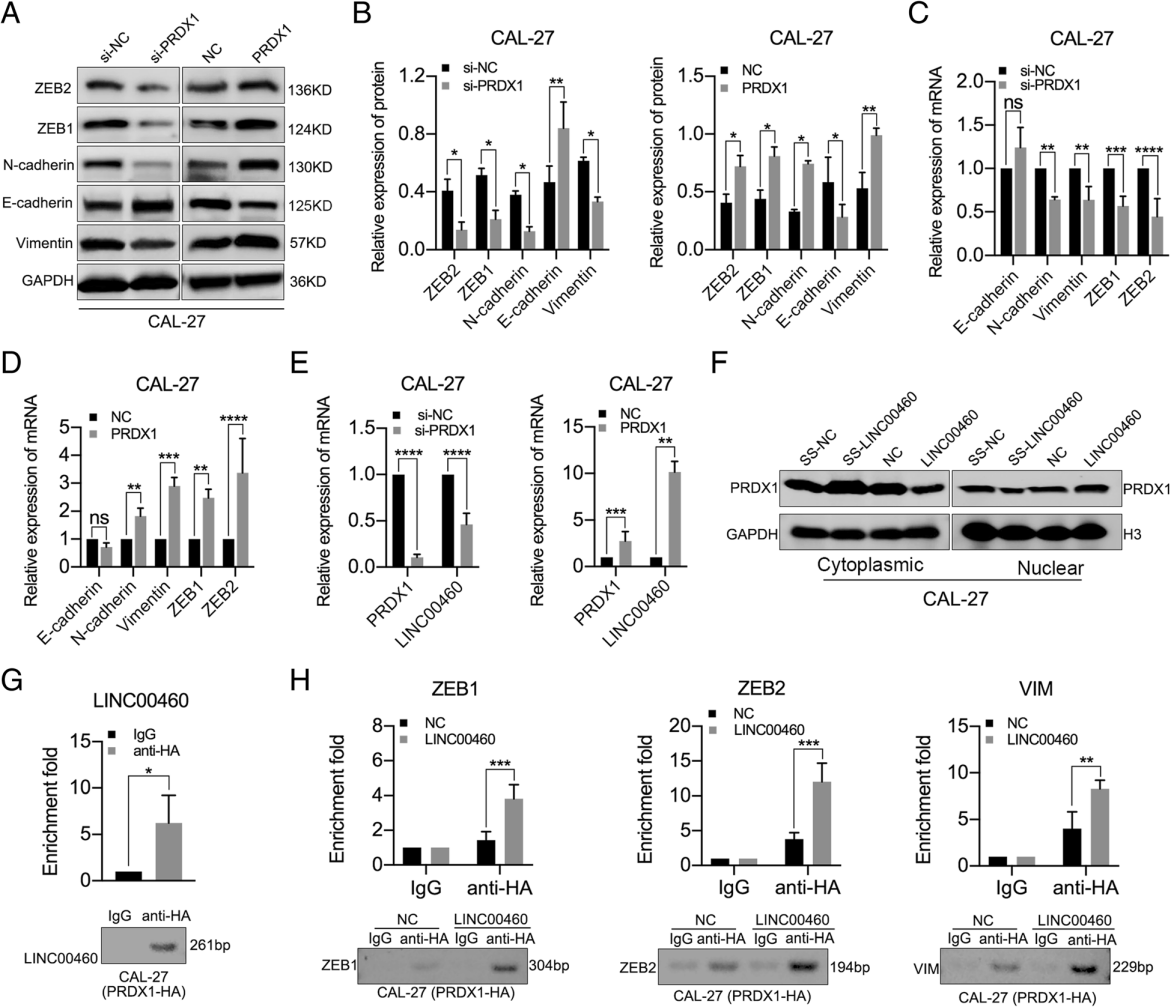
LINC00460 facilitated PRDX1 entry into the nucleus, and PRDX1 promoted the transcription of LINC00460 and EMT-related genes. **a**, **b** The expression of EMT markers (E-cadherin, N-cadherin, Vimentin, ZEB1 and ZEB2) was detected by Western blot analysis in CAL-27 cells when PRDX1 was knocked down or overexpressed. **c**, **d** The expression of EMT-associated genes (E-cadherin, N-cadherin, Vimentin, ZEB1 and ZEB2) was detected by qRT-PCR analysis in CAL-27 cells when PRDX1 was knocked down (**c**) or overexpressed (**d**). **e** qRT-PCR analysis of LINC00460 expression in CAL-27 cells when PRDX1 was knocked down or overexpressed. **f** The protein levels of PRDX1 in nuclear and cytoplasmic fractions were analyzed by Western blotting in CAL-27 cells transfected with SS-LINC00460 or LINC00460 vector. **g** ChIP-PCR analysis of anti-HA- or IgG- immunoprecipitated LINC00460 promoter fragments from CAL-27 cells stably transduced with PRDX1-HA. **h** ChIP-PCR analysis of anti-HA- or IgG- immunoprecipitated ZEB1, ZEB2 and VIM promoter fragments from CAL-27 cells stably transduced with PRDX1-HA when LINC00460 was overexpressed (LINC00460) or not overexpressed (NC). *p < 0.05, **p < 0.01, ***p < 0.001, ****p < 0.0001, ns: no significance

PRDX1 knockdown significantly increased the levels of E-cadherin and decreased the levels of N-cadherin, Vimentin, ZEB1 and ZEB2 in CAL-27 and HN30 cells, whereas the levels of E-cadherin were decreased and the levels of N-cadherin, Vimentin, ZEB1 and ZEB2 were increased in CAL-27 and HN30 cells stably transduced with PRDX1, as determined by Western blot analysis (Fig. 5a, b and Additional file 11: Figure S6A, B). PRDX1 knockdown significantly decreased the levels of N-cadherin, Vimentin, ZEB1 and ZEB2 in CAL-27 (Fig. 5c) and HN30 cells (Additional file 11: Figure S6C), whereas PRDX1 overexpression significantly increased the levels of N-cadherin, Vimentin, ZEB1 and ZEB2 in CAL-27 (Fig. 5d) and HN30 cells (Additional file 11: Figure S6D), as determined by qRT-PCR assays. Moreover, the expression of LINC00460 decreased when PRDX1 was silenced and increased when PRDX1 was overexpressed in CAL-27 (Fig. 5e) and HN30 cells (Additional file 11: Figure S6E), as determined by qRT-PCR. However, the abnormal expression of LINC00460 failed to affect the mRNA levels (Additional file 11: Figure S6F and G) and protein levels (Additional file 11: Figure S6H) of PRDX1. Importantly, we observed that PRDX1 levels were enriched in the nucleus and decreased in the cytoplasm when LINC00460 was overexpressed, suggesting that LINC00460 facilitated PRDX1 entry into the nucleus (Fig. 5f and Additional file 11: Figure S6I). To further clarify how PRDX1 promoted the expression of LINC00460 and EMT-related genes, ChIP assays were performed in CAL-27 cells transduced with PRDX1-HA (Additional file 11: Figure S6J). Specific primers for the 1000 bp upstream regions of the promoters of LINC00460, ZEB1, ZEB2 and VIM were designed (Additional file 11: Figure S6K), and all the primers had good specificity (Additional file 11: Figure S6L). From the results of ChIP assays, PRDX1 was enriched in the LINC00460 promoter fragment (Fig. 5g). Moreover, compared with NC group, overexpression of LINC00460 can effectively promote the enrichment of PRDX1 in promoter regions of ZEB1, ZEB2 and VIM genes (Fig. 5h). It showed that PRDX1 promoted the transcription of LINC00460 and EMT-related genes (such as ZEB1, ZEB2 and VIM) through enrichment in gene promoters in the nucleus.

### PRDX1 mediated the function of LINC00460 to promote the proliferation and metastasis of HNSCC cells

**Fig. 6:**
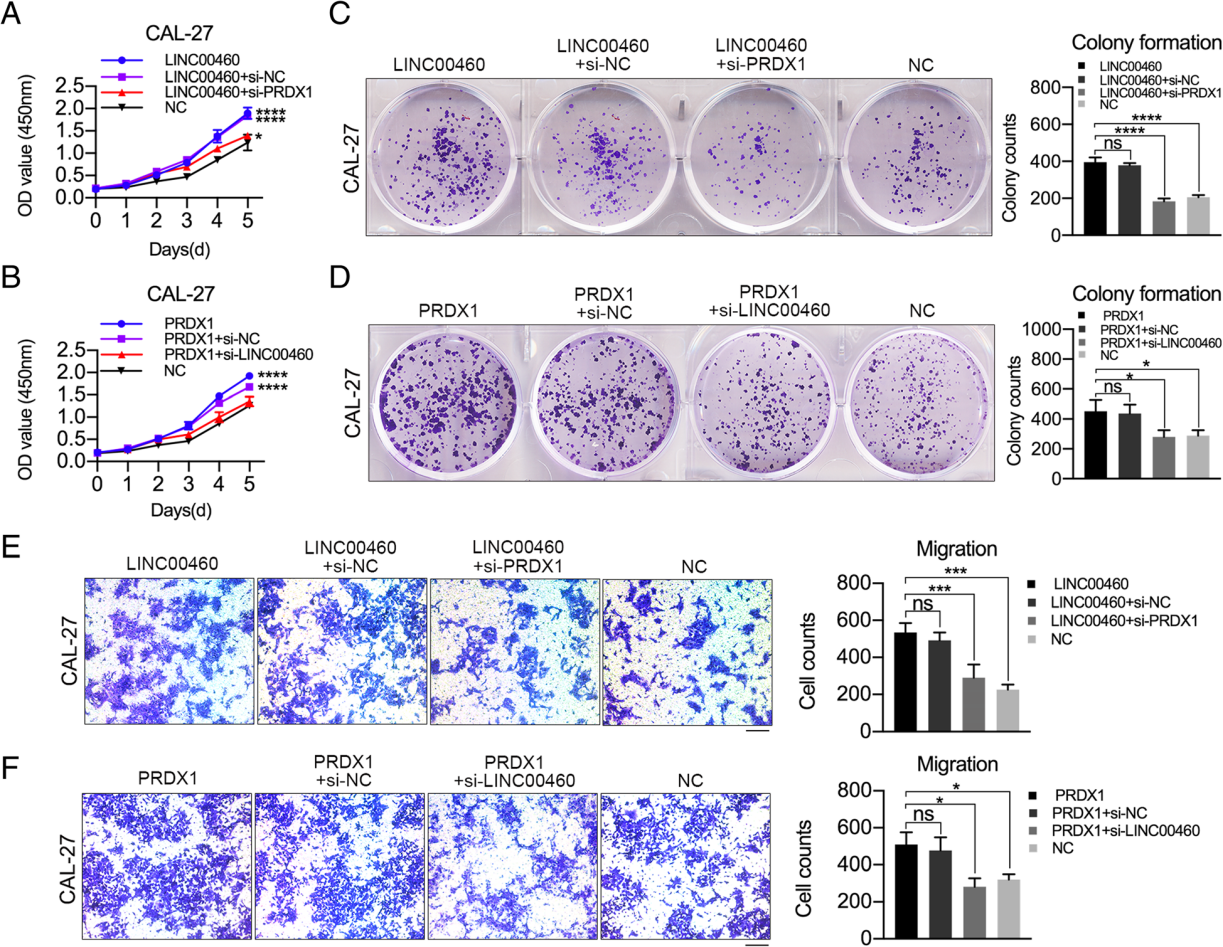
PRDX1 mediated the function of LINC00460 to promote the proliferation and metastasis of HNSCC cells. **a**, **b** The growth curves were detected by CCK-8 assay when PRDX1 was knocked down in CAL-27 cells stably transduced with LINC00460 (**a**) and when LINC00460 was knocked down in CAL-27 cells stably transduced with PRDX1 vector (**b**). **c**, **d** The colonizing abilities were detected by colony formation assays when PRDX1 was knocked down in CAL-27 cells stably transduced with LINC00460 (**c**) and when LINC00460 was knocked down in CAL-27 cells stably transduced with PRDX1 vector (**d**). **e**, **f** The migration abilities were assessed by transwell assays when PRDX1 was knocked down in CAL-27 cells stably transduced with LINC00460 (**e**) and when LINC00460 was knocked down in CAL-27 cells stably transduced with PRDX1 vector (**f**). *p < 0.05, ***p < 0.001, ****p < 0.0001, ns: no significance

Silencing of PRDX1 using si-PRDX1–1 (si-PRDX1) in CAL-27 cells significantly blocked the ability of LINC00460 to promote cell proliferation (Fig. 6a), colony formation (Fig. 6c) and migration assays (Fig. 6e). Knockdown of LINC00460 using si-LINC00460–1 (si-LINC00460) in CAL-27 cells also dramatically suppressed the ability of PRDX1 to promote cell proliferation (Fig. 6b), colony formation (Fig. 6d) and migration (Fig. 6f). These results showed that LINC00460 affected HNSCC cell proliferation and migration in a PRDX1-dependent manner.

### Upregulation of LINC00460 and PRDX1 correlated with poor clinicopathologic features in HNSCC patients

**Fig. 7:**
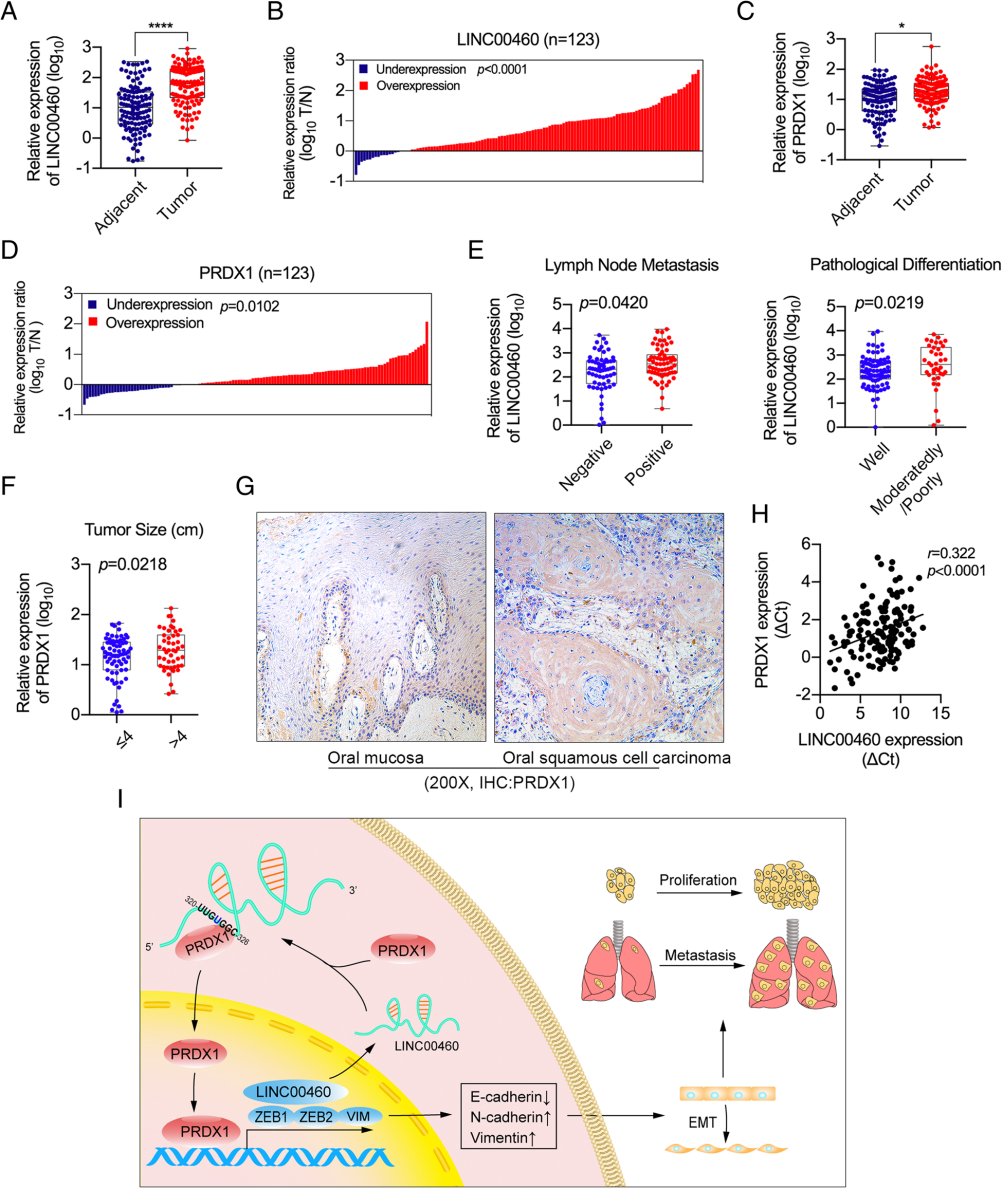
Upregulation of LINC00460 and PRDX1 was correlated with poor clinicopathologic features in HNSCC patients. **a** The relative expression of LINC00460 in HNSCC tissues (Tumor) and their adjacent normal tissues (Adjacent) was detected by qRT-PCR. ****p < 0.0001, *n* = 123. **b** LINC00460 expression was classified into two groups according to the expression ratio (T/N) of tumor tissue (T) to adjacent normal tissue (N) in HNSCC tissue. **c** The relative expression of PRDX1 in HNSCC tissues (Tumor) and their adjacent normal tissues (Adjacent) was detected by qRT-PCR. *p < 0.05, *n* = 123. **d** PRDX1 expression was classified into two groups according to the expression ratio (T/N) of HNSCC tumor tissue (T) to adjacent normal tissue (N). **e** The associations between the relative expression of LINC00460 and lymph metastasis/pathological differentiation were investigated in patients with HNSCC. *p* < 0.05. **f** The association between the relative expression of PRDX1 and tumor size was investigated in patients with HNSCC. *p* < 0.05. **g** Images of IHC staining of PRDX1 in normal oral mucosa and oral squamous cell carcinoma are shown. **h** Pearson correlation analysis of the expression of LINC00460 and PRDX1 in HNSCC tissue. *p* < 0.0001, *r* = 0.322, *n* = 143. **i** A proposed model illustrating the modulatory role of LINC00460 and PRDX1 in regulating HNSCC cell proliferation and metastasis

The expression of LINC00460 and PRDX1 was measured by qRT-PCR in 123 paired HNSCC tissues and adjacent normal tissues. The results clearly demonstrated that LINC00460 levels in HNSCC tissues were significantly higher than those in adjacent normal tissues (Fig. 7a and b), similar to the findings for PRDX1 (Fig. 7c and d). LINC00460 expression was positively associated with the pathological differentiation of tumors and with lymph node metastasis (*p* < 0.05) (Fig. 7e and Table 1), whereas PRDX1 expression was positively associated with tumor size (*p* < 0.05) (Fig. 7f). No significant associations between LINC00460/PRDX1 expression and other clinicopathologic features (sex, age, tumor–node–metastasis [TNM] stage, tumor site, local invasion, tumor type, etc.) were observed (Additional file 12: Figure S7A and B). The expression of PRDX1 in tumor tissue was significantly higher than that in normal oral mucosa, as determined by IHC (Fig. 7g). Furthermore, LINC00460 expression was positively correlated with PRDX1 expression in HNSCC tissues (Fig. 7h).

**Table 1:** Relationship between LINC00460 level and clinicopathologic features (*N* = 123)

Characteristics	No. of Patients		LINC00460 ΔCt^a^ Mean ± SD	Non-parametric test value	*p* value
	No.	%			
Age (years)					
≥ 60	81	65.85	8.553 ± 2.175	Z = 0.277	0.782
< 60	42	34.15	8.607 ± 2.906		
Gender					
Male	84	68.29	8.645 ± 2.758	Z = 0.386	0.700
Female	39	31.71	8.412 ± 1.555		
Tumor Size (cm)					
≤ 4	73	59.35	8.745 ± 2.548	Z = 0.608	0.543
> 4	50	40.65	8.319 ± 2.268		
Lymph Node Metastasis					
pN1 to pN3	63	51.22	8.022 ± 2.210	Z = 2.436	**0.015** ^*^
pN0	60	48.78	9.231 ± 2.635		
TNM Stage					
I	11	8.94	9.991 ± 3.331	H = 4.052	0.256
II	28	22.76	9.087 ± 2.559		
III	19	15.45	8.147 ± 2.719		
IV	65	52.85	8.233 ± 2.028		
Pathological Differentiation					
Well	85	69.11	8.810 ± 2.122	Z = 2.094	**0.036** ^*^
Moderately/poorly	38	30.89	8.038 ± 2.990		
Tumor Site					
Tongue	42	34.15	8.812 ± 1.928	H = 3.411	0.492
Gingival	36	29.27	8.293 ± 2.304		
Cheek	25	20.32	8.122 ± 2.246		
Floor of Mouth	6	4.88	8.802 ± 1.548		
Oropharynx	14	11.38	8.775 ± 3.268		
Local Invasion					
No	87	70.73	8.550 ± 2.604	Z = 0.183	0.854
Yes	36	29.27	8.624 ± 2.011		
Tumor Type					
Primary	105	85.37	8.723 ± 2.493	Z = 1.782	0.075
Recurrence	18	14.63	7.684 ± 1.907		

*Abbreviations*: *SD* standard deviation, *pN* pathological lymph node status

^a^The ΔCt value was determined by subtracting the GAPDH Ct value from the LINC00460 Ct value. Smaller ΔCt values indicate higher expression. *p<0.05

## Discussion

EMT is usually associated with tumor initiation, malignant progression, cell migration, tumor metastasis, etc. and is often defined by downregulated expression of epithelial markers (such as E-cadherin) and increased expression of mesenchymal markers (such as N-cadherin and Vimentin) [41]. Moreover, EMT-associated transcription factors (TFs), such as SNAI (SNAI1/Snail and SNAI2/Slug), ZEB (ZEB1 and ZEB2), and TWIST (TWIST1 and TWIST2) nuclear proteins, can repress E-cadherin expression and regulate the EMT process via different signaling pathways [42]. LncRNAs have been revealed to play essential roles in regulating the functions of EMT, so they are often considered promising biomarkers and therapeutic targets for EMT and metastasis [43].

Some previous studies have suggested that LINC00460 primarily affects cell invasion and migration in such cancers as esophageal cancers [21], epithelial ovarian cancer [27], colorectal cancer [23, 24] and gastric cancer [28]. It has been reported that LINC00460 induces EMT in lung cancer cells [16, 17]. Our study demonstrated that LINC00460 significantly enhanced cell proliferation, metastasis and the EMT phenotype in HNSCC cells. Moreover, the functions of LINC00460 in A549 and HeLa cells were consistent with those observed for HNSCC cells in our study and previous studies [18]. Owing to the lack of invasive HNSCC cell lines for a small-animal pulmonary metastasis model or EMT induction model, other highly invasive cell lines were usually used to replace HNSCC cell lines, such as MDA-MB-231 cells [44, 45], A549 cells [46], etc., which can partially reveal the metastasis characteristics of HNSCC cells. Since a pulmonary metastasis model by tail vein injection of A549 cells was successfully established [47–49], and the similar biological characteristics between A549 cells and HNSCC cells were also demonstrated according to previous studies [50–54], we finally used A549 cells for animal pulmonary metastasis assay to verify the in vivo functions of LINC00460. Though A549 cells transduced with LINC00460 cannot completely represent the pro-metastatic characteristics of LINC00460 for HNSCC cells, it is still valuable that A549 cells was used for the pulmonary metastasis model of HNSCC cells.

Since LINC00460 is primarily localized in the cytoplasm, some studies have shown that LINC00460 associates with a number of biomolecules, such as TFs, mRNAs, miRNAs and RBPs, to affect cancer development [15]. The previously reported mechanisms of LINC00460 in cancers are highly variable and contradictory. Some studies have investigated the effects of miRNAs on LINC00460 and its functions, revealing that LINC00460 promotes cell proliferation and migration by upregulating the expression of the miR-149-5p-targeted genes IL6 in nasopharyngeal carcinoma [25] and CUL4A in colorectal cancer [23], by targeting miR-342-3p/KDM2a in gastric cancer [28], by regulating miR-338-3p in epithelial ovarian cancer [27], by targeting miR-302c-5p/FOXA1 in human lung adenocarcinoma [18], by sponging miR-613 in papillary thyroid carcinoma [26], and by targeting miR-539/MMP-9 in meningioma [31]. In HNSCC cells, LINC00460 affects STC2 and promotes autophagy by regulating miRNA-206 [35]. Other studies have confirmed that LINC00460 can regulate gene expression through interactions with RBPs. One study reported that LINC00460 interacts with hnRNPK to promote EMT and cell migration in lung cancer cells [16]. The results of other studies demonstrated that LINC00460 exerts its oncogenic effects via the LINC00460/EZH2/KLF2 signaling axis in colorectal cancer cells [23] and showed that CBP/P300 binds to the LINC00460 promoter to activate LINC00460 transcription via histone acetylation to promote carcinogenesis in esophageal cancer cells [21]. However, the precise mechanisms by which LINC00460 affects cancer cell development and progression need to be further elucidated.

In our study, we discovered that PRDX1 was an RBP of LINC00460 and directly interacted with LINC00460 to affect proliferation, migration and EMT in HNSCC cells. PRDX1 is a major 2-Cys member of the peroxiredoxin family that plays important roles in cell proliferation, differentiation, and apoptosis under stress conditions and is associated with poor prognosis in cancers [55]. PRDX1 has previously been found to be an RBP [56, 57], and predicted binding sites for LINC00460 and PRDX1 are listed in the PRIdictor database. When the predicted binding sites (K at aa 120 of PRDX1 or nucleotides positions 320–326 of LINC00460) were mutated respectively, the binding capacity between PRDX1 and LINC00460 decreased, suggesting that the predicted binding sites played an important role in maintaining the binding of PRDX1 and LINC00460.

In our study, after PRDX1 knockdown in LINC00460-overexpressing cells or LINC00460 knockdown in PRDX1-overexpressing cells, cell proliferation and migration were significantly suppressed. When LINC00460 or PRDX1 was knocked down or overexpressed in HNSCC cells, the expression of EMT-associated genes were significantly altered. Furthermore, the expression of LINC00460 increased when PRDX1 was upregulated and decreased when PRDX1 was knocked down in HNSCC cells. PRDX1 was also enriched in the nucleus when LINC00460 was overexpressed, although total PRDX1 protein levels did not change. These findings above suggested that LINC00460 physically interacted with PRDX1 and facilitated PRDX1 entry into the nucleus. The results of ChIP assays showed that PRDX1 was enriched in the LINC00460 promoter fragment from nucleotide positions − 24 to − 284 upstream of the transcription start site (TSS). Moreover, overexpression of LINC00460 can enhance the enrichment of PRDX1 in the ZEB1 promoter fragment from nucleotide positions − 283 to − 586 upstream of TSS, the ZEB2 promoter fragment from nucleotide positions − 103 to − 296 upstream of TSS, and the VIM promoter fragment from nucleotide positions − 551 to − 779 upstream of TSS. PRDX1 promoted the transcription of LINC00460 and EMT-associated genes through enrichment in gene promoters in the nucleus. PRDX1 is an antioxidant that regulates cell growth, differentiation, apoptosis, and other functions and can affect gene regulation by associating with various TFs, including c-Myc, NF-кB, and AR in the nucleus [55, 58, 59]. These results suggested that the enhancement of LINC00460 transcription was promoted by PRDX1 possibly through its interaction with TFs. As a result, LINC00460 effectively induced the HNSCC cell EMT in a PRDX1-dependent manner, and PRDX1 mainly mediated the EMT-promoting effect of LINC00460 (Fig. 7i).

In our previous study, LINC00460 was found to be associated with the prognosis of HNSCC [13]. LINC00460 is positively correlated with advanced TNM stages and lymph node metastasis in papillary thyroid carcinoma [26] and with invasion depth and TNM stage in colorectal cancer [24]. Our study showed that the expression of LINC00460 was associated with lymph node metastasis and pathological differentiation, which is consistent with the results of esophageal cancer [21]. Aberrant PRDX1 expression occurs in numerous cancers [55]. PRDX1 has been reported to be an independent prognostic factor for disease recurrence and reduced survival in non–small-cell lung cancer [60] and gastric cancer [61]. PRDX1 was overexpressed in oral leukoplakia and oral cancers [62], and associated with local recurrence, which may be clinically useful in guiding treatment for HNSCC patients [63, 64]. Our results also showed that the expression of PRDX1 was associated with tumor size, and was positively correlated with the expression of LINC00460. Thus, the association between LINC00460 and PRDX1 may be a biomarker for accurate prognosis and a potential target for cancer therapy in the context of HNSCC.

## Conclusions

In this study, LINC00460 was shown to promote HNSCC cell proliferation and metastasis in vitro and in vivo. LINC00460 primarily localized within the cytoplasm, physically interacted with PRDX1 and facilitated PRDX1 entry into the nucleus in HNSCC cells. In the nucleus, PRDX1 promoted the transcription of LINC00460 and EMT-related genes through enrichment in gene promoters in the nucleus. LINC00460 effectively induced the HNSCC cell EMT phenotype in a PRDX1-dependent manner, and PRDX1 mainly mediated the EMT-promoting effect of LINC00460. LINC00460 expression in HNSCC was correlated with lymph node metastasis and pathological differentiation, while PRDX1 expression was correlated with tumor size. LINC00460 and PRDX1 may serve as biomarkers for accurate prognostic prediction and as potential targets for cancer therapy in HNSCC patients.

## Additional files

Additional file 1: **Table S1.** The RRIDs of the cell lines used in this study. (DOCX 15 kb)

Additional file 2: **Table S2.** Sequences of the qRT-PCR primers. (DOCX 17 kb)

Additional file 3: **Table S3.** Smart Silencer and siRNA sequences. (DOCX 16 kb)

Additional file 4: **Table S4.** Sequences of the RIP primers for LINC00460. (DOCX 16 kb)

Additional file 5: **Table S5.** Sequences of the ChIP promoter primers. (DOCX 16 kb)

Additional file 6: **Figure S1.** Effect of LINC00460 overexpression on A549 and HeLa cell proliferation and migration in vitro. (A) Construction of the LINC00460 lentiviral expression vector (LINC00460 vector). (B) The relative expression of LINC00460 in A549 and HeLa cells stably transduced with LINC00460 vector (LINC00460) was detected by qRT-PCR. (C) The effect of LINC00460 expression on cell proliferation was evaluated with A549 and HeLa cells stably transduced with LINC00460 by CCK-8 assays. (D) The colonizing abilities of A549 and HeLa cells stably transduced with LINC00460 were determined by colony formation assays. (E) The cell migration abilities of A549 and HeLa cells stably transduced with LINC00460 were determined by transwell assays. Scale bar: 1000 μm. *p < 0.05, **p < 0.01, ***p < 0.001, ****p < 0.0001. (TIF 2793 kb)

Additional file 7: **Figure S2.** LINC00460 induced cell EMT. (A) The differences in E-cadherin and Vimentin expression in CAL-27 cells when LINC00460 was overexpressed were detected by immunofluorescence assays. (B) The expression of E-cadherin, N-cadherin and vimentin was detected by Western blot analysis when LINC00460 was overexpressed in A549 and HeLa cells. (TIF 510 kb)

Additional file 8: **Figure S3.** The silencing efficiencies of si-LINC00460–1, si-LINC00460–2 and si-LINC00460–3 in CAL-27 cells were determined by qRT-PCR analysis. ****p < 0.0001. (TIF 80 kb)

Additional file 9: **Figure S4.** PRDX1 was identified as an RBP of LINC00460. (A) The process of RNA pull-down assays performed in this study. (B) The results of mass spectrometry analysis following RNA pull-down assays using negative and LINC00460 probes in CAL-27 cells stably transduced with LINC00460. (C) PRDX1 was one of the proteins selected from the mass spectrometry results. (D) Construction of the PRDX1 lentiviral expression vector (PRDX1-HA). (E, F) The relative expression of PRDX1 in HN30 cells stably transduced with the PRDX1-HA was detected by Western blot analysis (E) and qRT-PCR (F). (G) qRT-PCR analysis of LINC00460 enriched with anti-HA in HN30 cells stably transduced with PRDX1-HA in RIP assays. (H) The predicted LINC00460-binding site of PRDX1 was obtained from the PRIdictor database (http://bclab.inha.ac.kr/pridictor). (I) qRT-PCR analysis of LINC00460 enriched by anti-HA in HN30 cells transfected with PRDX1-HA-WT and PRDX1-HA-Mut vectors in RIP assays. (J, K) The predicted PRDX1-binding sites of LINC00460 were obtained from the PRIdictor database. (TIF 4579 kb)

Additional file 10: **Figure S5.** PRDX1 affected cell proliferation and migration in HN30 cells. (A) The silencing efficiency of si-PRDX1 in HN30 cells was detected by qRT-PCR. (B, C) The silencing efficiency of si-PRDX1 in CAL-27 (B) and HN30 cells (C) was detected by Western blot analysis. (D) The effect of PRDX1 expression on cell proliferation was evaluated with HN30 cells transfected with si-PRDX1 by CCK-8 assays. (E) The colonizing ability of HN30 cells transfected with si-PRDX1 was determined by colony formation assays. (F) The cell migration abilities of HN30 cells transfected with si-PRDX1 were determined by transwell assays. *p < 0.05, **p < 0.01, ***p < 0.001, ****p < 0.0001. (TIF 3167 kb)

Additional file 11: **Figure S6.** PRDX1 affected EMT in HNSCC cells and promoted the transcription of LINC00460 and EMT-related genes. (A, B) The expression of EMT markers (E-cadherin, N-cadherin, Vimentin, ZEB1 and ZEB2) was detected by Western blot analysis when PRDX1 was knocked down or overexpressed in HN30 cells. (C, D) The expression of EMT-related genes (E-cadherin, N-cadherin, Vimentin, ZEB1 and ZEB2) was detected by qRT-PCR when PRDX1 was knocked down (C) or overexpressed (D) in HN30 cells. (E) qRT-PCR analysis of LINC00460 expression in HN30 cells when PRDX1 was knocked down or overexpressed. (F, G) qRT-PCR analysis of PRDX1 expression when LINC00460 was knocked down (F) or overexpressed (G) in HNSCC cells. (H) The protein level of PRDX1 was analyzed by Western blotting when LINC00460 was knocked down or overexpressed in HNSCC cells. (I) The protein levels of PRDX1 in nuclear and cytoplasmic fractions were analyzed by Western blotting in HN30 cells transfected with SS-LINC00460 and in SCC-9 cells transduced with LINC00460. (J) The process of the ChIP assay performed in our study. (K) The specific primers for the promoter region were designed for ChIP assays. TSS, Transcription Start Site. (L) The ChIP primers provided specific amplification. *p < 0.05, **p < 0.01, ***p < 0.001, ****p < 0.0001, ns: no significance. (TIF 1158 kb)

Additional file 12: **Figure S7.** Analysis of the association between LINC00460 and PRDX1 expression and clinical significance in HNSCC tissues. (A) The associations between the relative expression of LINC00460 and clinical parameters were investigated in patients with HNSCC (*p* > 0.05). (B) The associations between the relative expression of PRDX1 and clinical parameters were investigated in patients with HNSCC (*p* > 0.05). (TIF 1013 kb)

## Data Availability

The datasets supporting the conclusions of this article are included within the article and its additional files.

## Abbreviations

aa: Amino acid
ChIP: Chromatin immunoprecipitation
EMT: Epithelial–mesenchymal transition
FISH: Fluorescence in situ hybridization
HNSCC: Head and neck squamous cell carcinoma
HRP: Horseradish peroxidase
IgG: Immunoglobulin G
lncRNA: Long noncoding RNA
mRNA: Messenger RNA
NC: Negative control
PRDX1: Peroxiredoxin-1
qRT-PCR: Quantitative real-time polymerase chain reaction
RBP: RNA-binding protein
RIP: RNA immunoprecipitation
siRNA: Small interfering RNA
TCGA: *The Cancer Genome Atlas*
TF: Transcription factor
TNM: Tumor–node–metastasis
TSS: Transcription start site
VIM: Vimentin
ZEB1: Zinc-finger E-box-binding homeobox 1
ZEB2: Zinc-finger E-box-binding homeobox 2
