# Supplementary material for: LncRNA LINC00460 promotes EMT in head and neck squamous cell carcinoma by facilitating peroxiredoxin-1 into the nucleus
Source: J Exp Clin Cancer Res. 2019 Aug 20;38:365. doi: 10.1186/s13046-019-1364-z (PMC6700841; doi:10.1186/s13046-019-1364-z)
Supplement: Supplementary file 1 — Table S1. The RRIDs of the cell lines used in this study. (DOCX 15 kb) [file 13046_2019_1364_MOESM1_ESM.docx]

**LncRNA LINC00460 promotes EMT in head and neck squamous cell carcinoma by facilitating Peroxiredoxin-1 into the nucleus**

**Additional file 1: Table S1.** The RRIDs of cell lines used in this study.

| **Cell Lines** | **ATCC Cat#** | **RRID** |
| --- | --- | --- |
| WSU-HN4 | N/A | CVCL_5515 |
| WSU-HN6 | N/A | CVCL_5516 |
| WSU-HN30 | N/A | CVCL_5525 |
| CAL-27 | CRL-2095 | CVCL_1107 |
| SCC-4 | CRL-1624 | CVCL_1684 |
| SCC-9 | CRL-1629 | CVCL_1685 |
| SCC-25 | CRL-1628 | CVCL_1682 |
| A549 | CCL-185 | CVCL_0023 |
| HeLa | CCL-2 | CVCL_0030 |

N/A, Not Applicable.
