# Supplementary material for: LncRNA LINC00460 promotes EMT in head and neck squamous cell carcinoma by facilitating peroxiredoxin-1 into the nucleus
Source: J Exp Clin Cancer Res. 2019 Aug 20;38:365. doi: 10.1186/s13046-019-1364-z (PMC6700841; doi:10.1186/s13046-019-1364-z)
Supplement: Supplementary file 2 — Table S2. Sequences of the qRT-PCR primers. (DOCX 17 kb) [file 13046_2019_1364_MOESM2_ESM.docx]

**Additional file 2: Table S2.** Sequences of qRT-PCR primers.

| **Primers** | | **Sequences (5'-3')** |
| --- | --- | --- |
| LINC00460 | Forward | GGCATTGTAGAAAGACTGAGCG |
|  | Reverse | TAGCATACGAATTTGGGTGGG |
| GAPDH | Forward | GAACGGGAAGCTCACTGG |
|  | Reverse | GCCTGCTTCACCACCTTCT |
| U6 | Forward | CTCGCTTCGGCAGCACATATACT |
|  | Reverse | ATTTGCGTGTCATCCTTGCGCA |
| TUG1 | Forward | TCCTTGTTTAGTGCATCTTTGCC |
|  | Reverse | TGAGTGGTTATTCTGATAGCCTGC |
| NEAT1 | Forward | GCATACGCAGCAGATCAGCAT |
|  | Reverse | CCCACAATATAGGCATTTACAAGG |
| MALAT1 | Forward | CCTAACCAGGCATAACACAGAAT |
|  | Reverse | CGAATGGCTTTGTCTCCGAA |
| BIRC5 | Forward | GCAATGTCTTAGGAAAGGAGATCA |
|  | Reverse | AGAGAAGCAGCCACTGTTACCA |
| E-cadherin | Forward | CGAGAGCTACACGTTCACGG |
|  | Reverse | GGGTGTCGAGGGAAAAATAGG |
| N-cadherin | Forward | TGCGGTACAGTGTAACTGGG |
|  | Reverse | GAAACCGGGCTATCTGCTCG |
| Vimentin | Forward | AGTCCACTGAGTACCGGAGAC |
|  | Reverse | CATTTCACGCATCTGGCGTTC |
| PRDX1 | Forward | CCACGGAGATCATTGCTTTCA |
|  | Reverse | AGGTGTATTGACCCATGCTAGAT |
| SNAI1 | Forward | TCGGAAGCCTAACTACAGCGA |
|  | Reverse | AGATGAGCATTGGCAGCGAG |
| SNAI2 | Forward | CGAACTGGACACACATACAGTG |
|  | Reverse | CTGAGGATCTCTGGTTGTGGT |
| ZEB1 | Forward | GATGATGAATGCGAGTCAGATGC |
|  | Reverse | ACAGCAGTGTCTTGTTGTTGT |
| ZEB2 | Forward | CAAGAGGCGCAAACAAGCC |
|  | Reverse | GGTTGGCAATACCGTCATCC |
