# Supplementary material for: LncRNA LINC00460 promotes EMT in head and neck squamous cell carcinoma by facilitating peroxiredoxin-1 into the nucleus
Source: J Exp Clin Cancer Res. 2019 Aug 20;38:365. doi: 10.1186/s13046-019-1364-z (PMC6700841; doi:10.1186/s13046-019-1364-z)
Supplement: Supplementary file 3 — Table S3. Smart Silencer and siRNA sequences. (DOCX 16 kb) [file 13046_2019_1364_MOESM3_ESM.docx]

**Additional file 3: Table S3.** Smart Silencer and siRNA sequences.

| **Product Name** | **Sequences** |
| --- | --- |
| LINC00460 Smart Silencer | AGACCTAATAGCCAATAAG |
|  | CCATGTGAAGTGTAGAACA |
|  | TGGTGACAATAACACTGTG |
|  | GGTACCCAGACATTGTTATG |
|  | ACCTTGACTACTGCTATAGA |
|  | TAACCTTGGAGTCCACGCCT |
| si-PRDX1-1 | CCATGAACATTCCTTTGGT |
| si-PRDX1-2 | GGAGATCATTGCTTTCAGT |
| si-PRDX1-3 | CAGCCTGTCTGACTACAAA |
| si-LINC00460-1 ^*^ | AGACCTAATAGCCAATAAG |
| si-LINC00460-2 | CCATGTGAAGTGTAGAACA |
| si-LINC00460-3 | TGGTGACAATAACACTGTG |

^*^ siRNA for cholesterol-conjugated LINC00460 siRNA for *in vivo* siRNA delivery and rescue experiment.
