# Supplementary material for: LncRNA LINC00460 promotes EMT in head and neck squamous cell carcinoma by facilitating peroxiredoxin-1 into the nucleus
Source: J Exp Clin Cancer Res. 2019 Aug 20;38:365. doi: 10.1186/s13046-019-1364-z (PMC6700841; doi:10.1186/s13046-019-1364-z)
Supplement: Supplementary file 4 — Table S4. Sequences of the RIP primers for LINC00460. (DOCX 16 kb) [file 13046_2019_1364_MOESM4_ESM.docx]

**Additional file 4: Table S4.** Sequences of RIP primers for LINC00460.

| **Primer No.** | | **Sequences (5'-3')** |
| --- | --- | --- |
| P1 | Forward | AGCCCTGTTAGAAATGCCTCA |
|  | Reverse | CAATGTCTGGGTACCTCCGAT |
| P2 | Forward | CAGACATTGTTATGAAACTCCGC |
|  | Reverse | CTTAGCCGAGAAGTGTGCATG |
| P3 | Forward | CATGCACACTTCTCGGCTAAG |
|  | Reverse | CCTCCCACACAATGGTCGTAA |
| P4 | Forward | AACGAAGGTTACGACCATTGTG |
|  | Reverse | CCCACGCTCAGTCTTTCTACAA |
| P5 | Forward | GGCATTGTAGAAAGACTGAGCG |
|  | Reverse | TAGCATACGAATTTGGGTGGG |
| P6 | Forward | CCCACCCAAATTCGTATGCTA |
|  | Reverse | CTGGTTTTGAGGACTCTGCCC |
| P7 | Forward | CTCAAAACCAGATAAGTGCCCG |
|  | Reverse | CACTTCACATGGTAGACGGTGC |
| P8 | Forward | CCCTCACCAGAACCCAGTTGT |
|  | Reverse | GATGGCTCAGGAAAAACAAACT |
| P9 | Forward | CTGTCATAGCTCCCCAAATAGACT |
|  | Reverse | GTGTTATTGTCACCATTTCAGAGG |
| P10 | Forward | GCTCCCCAAATAGACTAAGACATC |
|  | Reverse | ACACAGTGTTATTGTCACCATTTCA |
