# Supplementary material for: LncRNA LINC00460 promotes EMT in head and neck squamous cell carcinoma by facilitating peroxiredoxin-1 into the nucleus
Source: J Exp Clin Cancer Res. 2019 Aug 20;38:365. doi: 10.1186/s13046-019-1364-z (PMC6700841; doi:10.1186/s13046-019-1364-z)
Supplement: Supplementary file 5 — Table S5. Sequences of the ChIP promoter primers. (DOCX 16 kb) [file 13046_2019_1364_MOESM5_ESM.docx]

**Additional file 5: Table S5.** Sequences of promoter CHIP primers.

| **Gene** |  |  | **Sequences (5'-3')** |
| --- | --- | --- | --- |
| ZEB1 | 1 | Forward | TTTGGCATTGAGGATGAATGCAG |
|  |  | Reverse | ACTGAAACGTGACCGGAGTA |
|  | 2 | Forward | TCTCTATCAATAACTGCTACATTG |
|  |  | Reverse | CTTAAGGCAAGAAGCATCGG |
|  | 3 | Forward | GGCGATGACCGCTCATTTAGG |
|  |  | Reverse | GAGAGGCTAGAAGTTCCGCT |
| ZEB2 | 1 | Forward | ACTTGCTAAATTGCTGACTTCCAG |
|  |  | Reverse | ACCTCGGGGAGACCTCACTA |
|  | 2 | Forward | AGATCGGCCAACCGAGTGTT |
|  |  | Reverse | ATCCCGGGTCCTCAATCCAG |
|  | 3 | Forward | ACCTACACAATTTGATGTGCATCTC |
|  |  | Reverse | CTTTGGCATCATTATCCTCATCACT |
| VIM | 1 | Forward | GCAGCCTCCCAGTGAAAGAG |
|  |  | Reverse | AGGGGGTACTGCAGGTTACT |
|  | 2 | Forward | CCAAGTAACCTGCAGTACCC |
|  |  | Reverse | GCTGAGTACTTACCCGCCAA |
|  | 3 | Forward | TTATCTCCCTTGGCGGGTAAG |
|  |  | Reverse | AGGGTTCACGGTGATTTGTCT |
|  | 4 | Forward | GGCATTTCCCAGACAAATCACC |
|  |  | Reverse | GGTTTTTACCCTGGTGGAAGTCA |
|  | 5 | Forward | CCGCCAAAGATTCTGTCATTTGTG |
|  |  | Reverse | TGAAAATTCTGGGTGAAAGAGGAA |
