# Supplementary figures and images for: LncRNA LINC00460 promotes EMT in head and neck squamous cell carcinoma by facilitating peroxiredoxin-1 into the nucleus
Source: J Exp Clin Cancer Res. 2019 Aug 20;38:365. doi: 10.1186/s13046-019-1364-z (PMC6700841; doi:10.1186/s13046-019-1364-z)

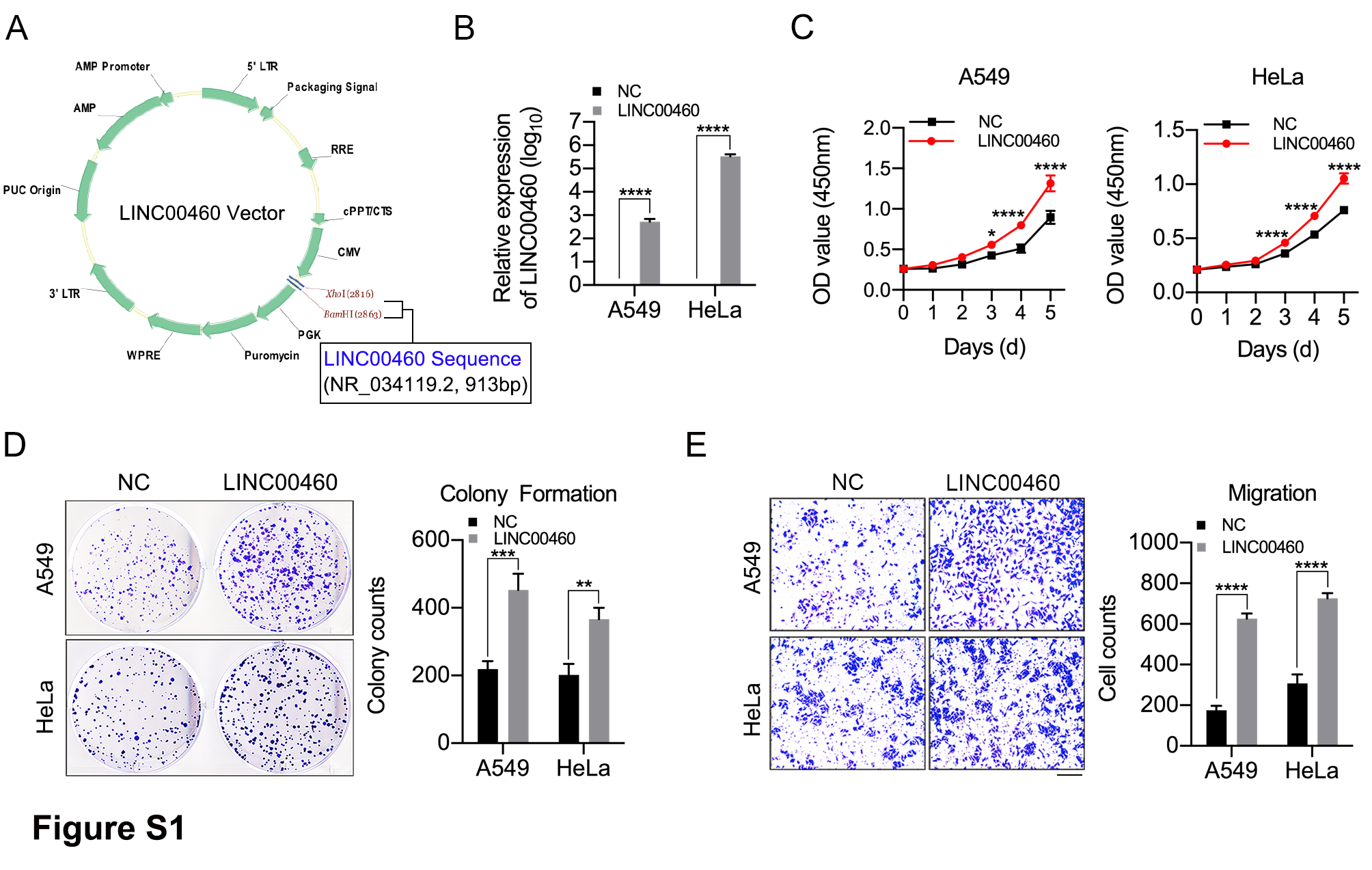

Supplement: Supplementary file 6 — Figure S1. Effect of LINC00460 overexpression on A549 and HeLa cell proliferation and migration in vitro. (A) Construction of the LINC00460 lentiviral expression vector (LINC00460 vector). (B) The relative expression of LINC00460 in A549 and HeLa cells stably transduced with LINC00460 vector (LINC00460) was detected by qRT-PCR. (C) The effect of LINC00460 expression on cell proliferation was evaluated with A549 and HeLa cells stably transduced with LINC00460 by CCK-8 assays. (D) The colonizing abilities of A549 and HeLa cells stably transduced with LINC00460 were determined by colony formation assays. (E) The cell migration abilities of A549 and HeLa cells stably transduced with LINC00460 were determined by transwell assays. Scale bar: 1000 μm. *p < 0.05, **p < 0.01, ***p < 0.001, ****p < 0.0001. (TIF 2793 kb) [file 13046_2019_1364_MOESM6_ESM.tif]

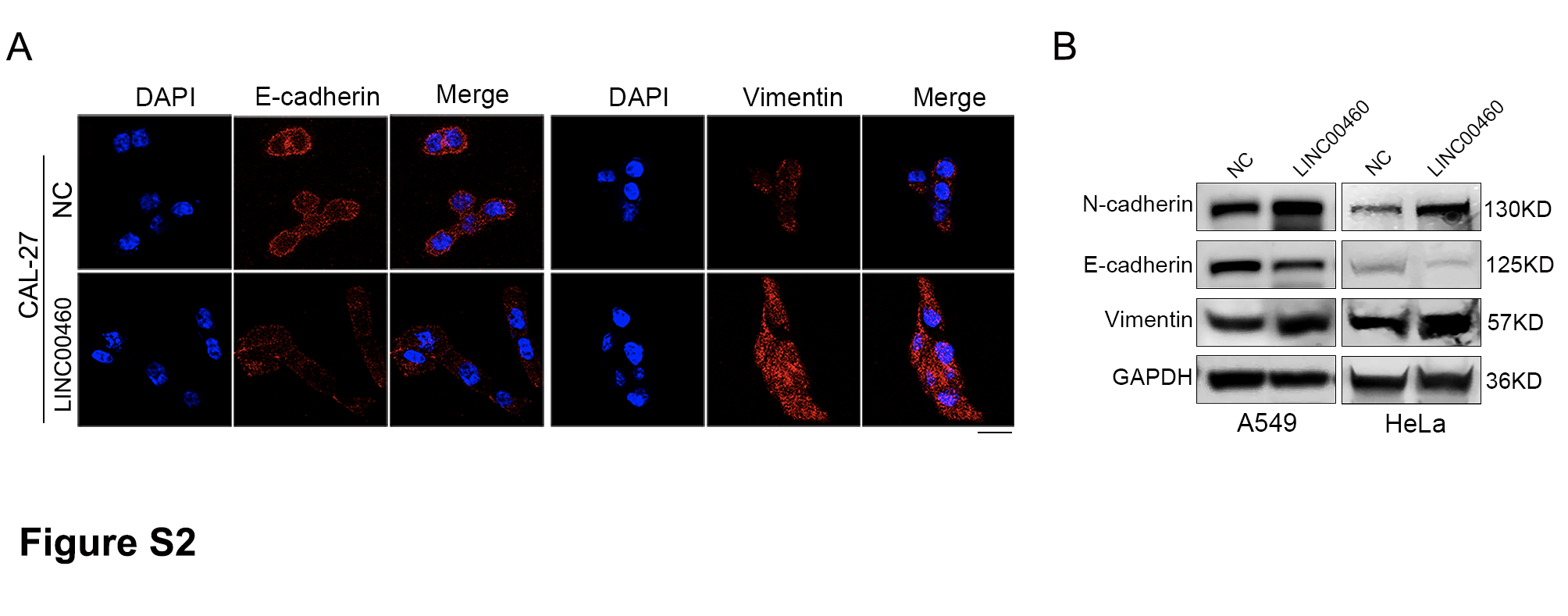

Supplement: Supplementary file 7 — Figure S2. LINC00460 induced cell EMT. (A) The differences in E-cadherin and Vimentin expression in CAL-27 cells when LINC00460 was overexpressed were detected by immunofluorescence assays. (B) The expression of E-cadherin, N-cadherin and vimentin was detected by Western blot analysis when LINC00460 was overexpressed in A549 and HeLa cells. (TIF 510 kb) [file 13046_2019_1364_MOESM7_ESM.tif]

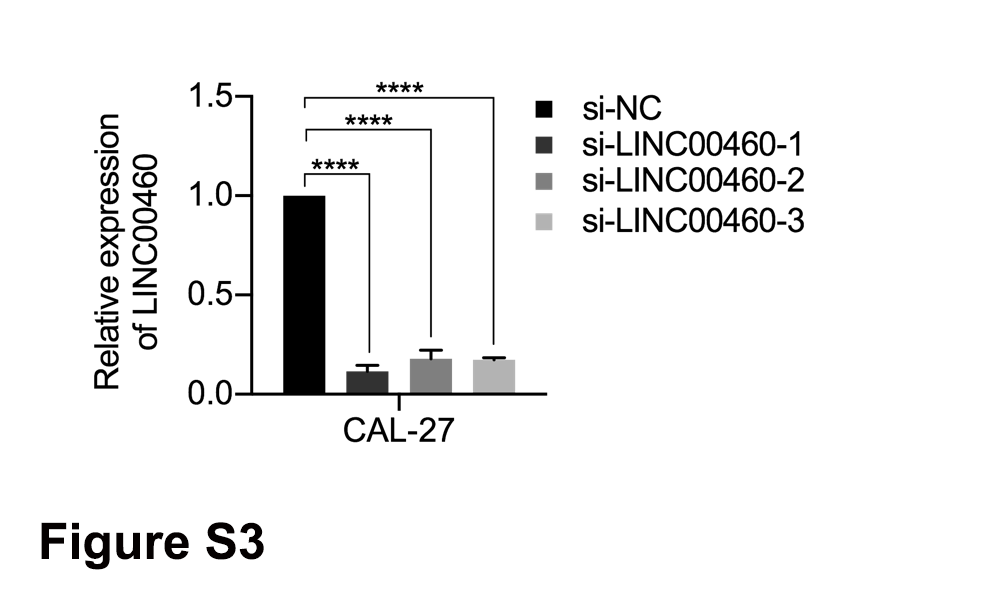

Supplement: Supplementary file 8 — Figure S3. The silencing efficiencies of si-LINC00460–1, si-LINC00460–2 and si-LINC00460–3 in CAL-27 cells were determined by qRT-PCR analysis. ****p < 0.0001. (TIF 80 kb) [file 13046_2019_1364_MOESM8_ESM.tif]

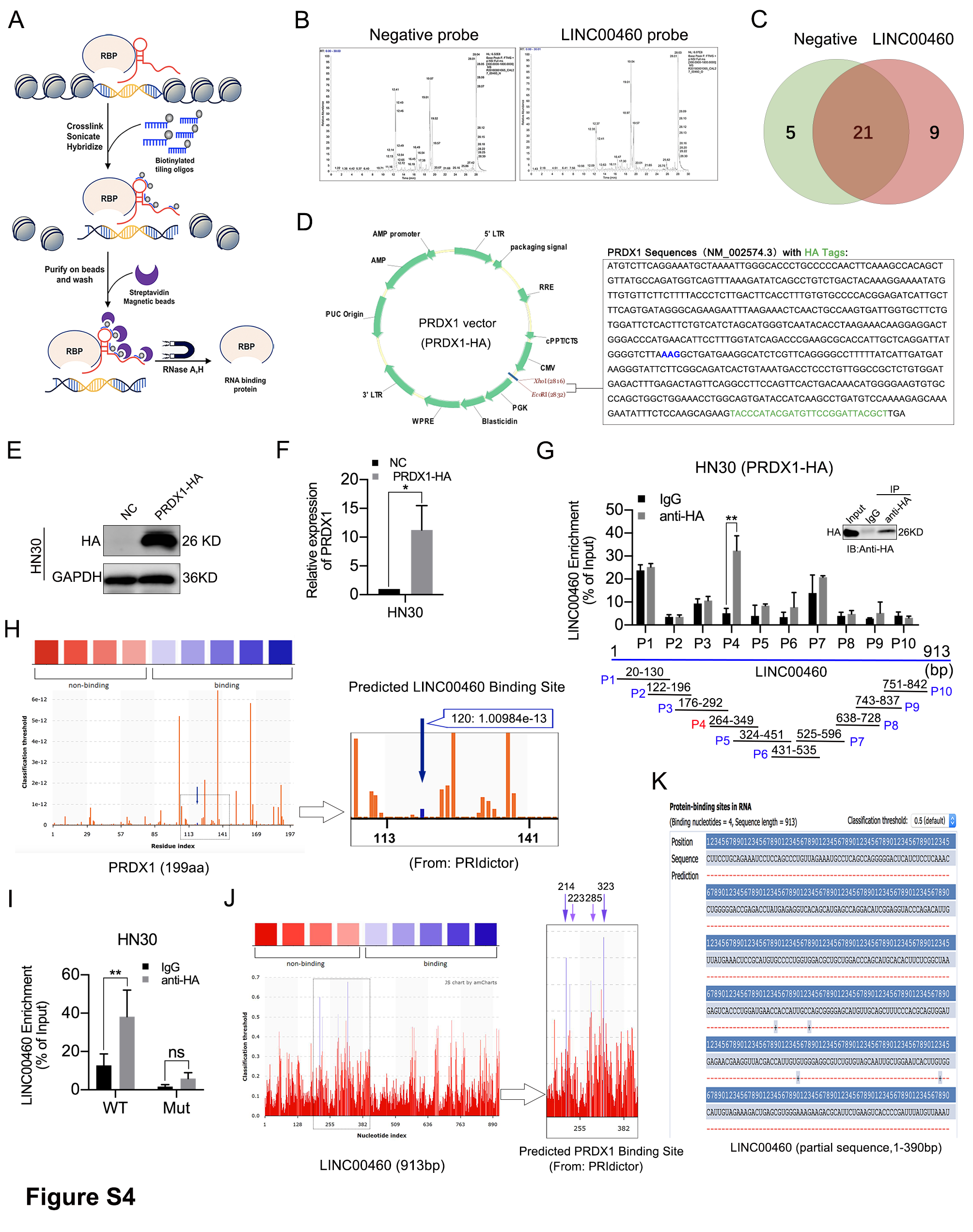

Supplement: Supplementary file 9 — Figure S4. PRDX1 was identified as an RBP of LINC00460. (A) The process of RNA pull-down assays performed in this study. (B) The results of mass spectrometry analysis following RNA pull-down assays using negative and LINC00460 probes in CAL-27 cells stably transduced with LINC00460. (C) PRDX1 was one of the proteins selected from the mass spectrometry results. (D) Construction of the PRDX1 lentiviral expression vector (PRDX1-HA). (E, F) The relative expression of PRDX1 in HN30 cells stably transduced with the PRDX1-HA was detected by Western blot analysis (E) and qRT-PCR (F). (G) qRT-PCR analysis of LINC00460 enriched with anti-HA in HN30 cells stably transduced with PRDX1-HA in RIP assays. (H) The predicted LINC00460-binding site of PRDX1 was obtained from the PRIdictor database (http://bclab.inha.ac.kr/pridictor). (I) qRT-PCR analysis of LINC00460 enriched by anti-HA in HN30 cells transfected with PRDX1-HA-WT and PRDX1-HA-Mut vectors in RIP assays. (J, K) The predicted PRDX1-binding sites of LINC00460 were obtained from the PRIdictor database. (TIF 4579 kb) [file 13046_2019_1364_MOESM9_ESM.tif]

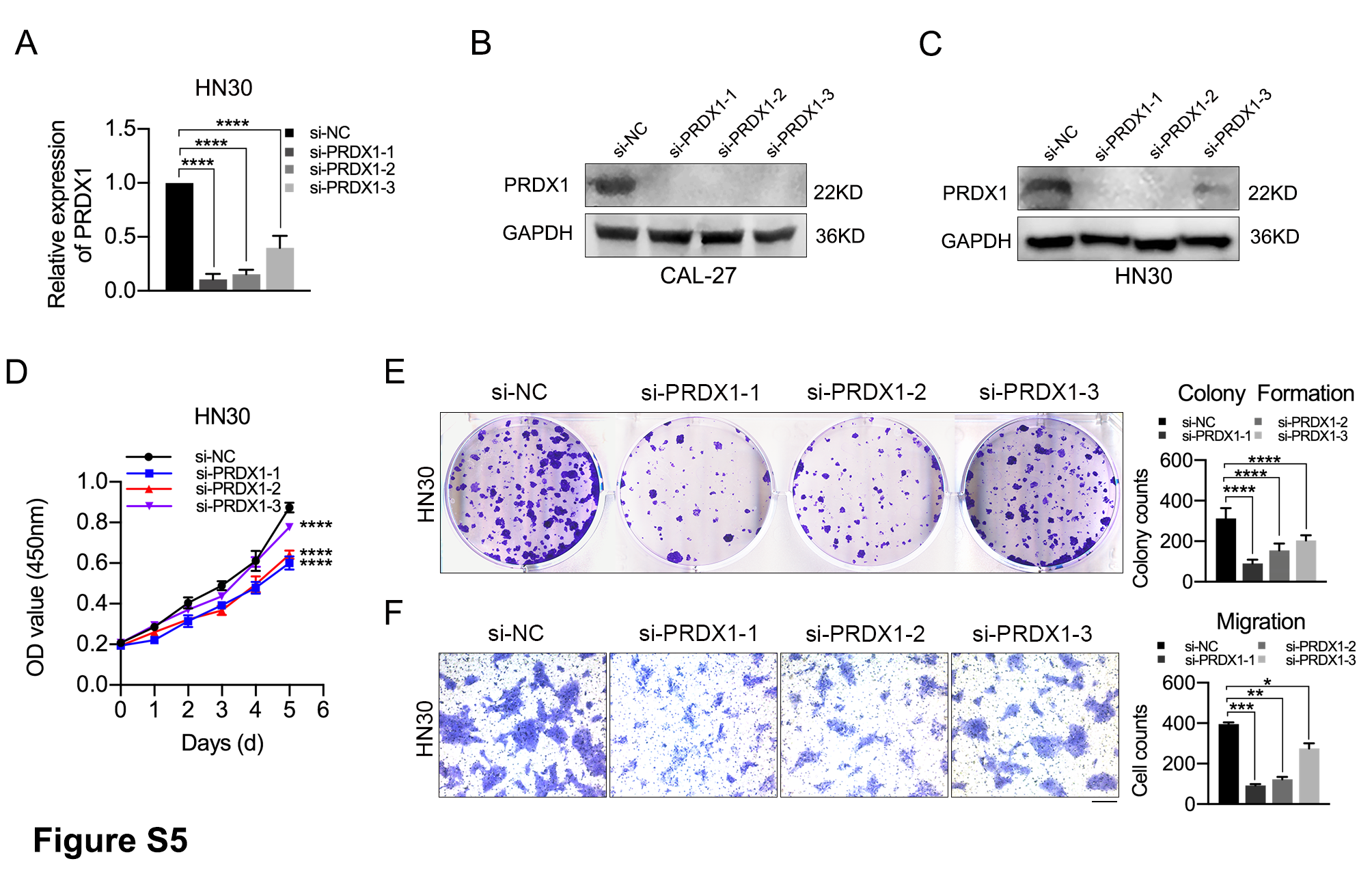

Supplement: Supplementary file 10 — Figure S5. PRDX1 affected cell proliferation and migration in HN30 cells. (A) The silencing efficiency of si-PRDX1 in HN30 cells was detected by qRT-PCR. (B, C) The silencing efficiency of si-PRDX1 in CAL-27 (B) and HN30 cells (C) was detected by Western blot analysis. (D) The effect of PRDX1 expression on cell proliferation was evaluated with HN30 cells transfected with si-PRDX1 by CCK-8 assays. (E) The colonizing ability of HN30 cells transfected with si-PRDX1 was determined by colony formation assays. (F) The cell migration abilities of HN30 cells transfected with si-PRDX1 were determined by transwell assays. *p < 0.05, **p < 0.01, ***p < 0.001, ****p < 0.0001. (TIF 3167 kb) [file 13046_2019_1364_MOESM10_ESM.tif]

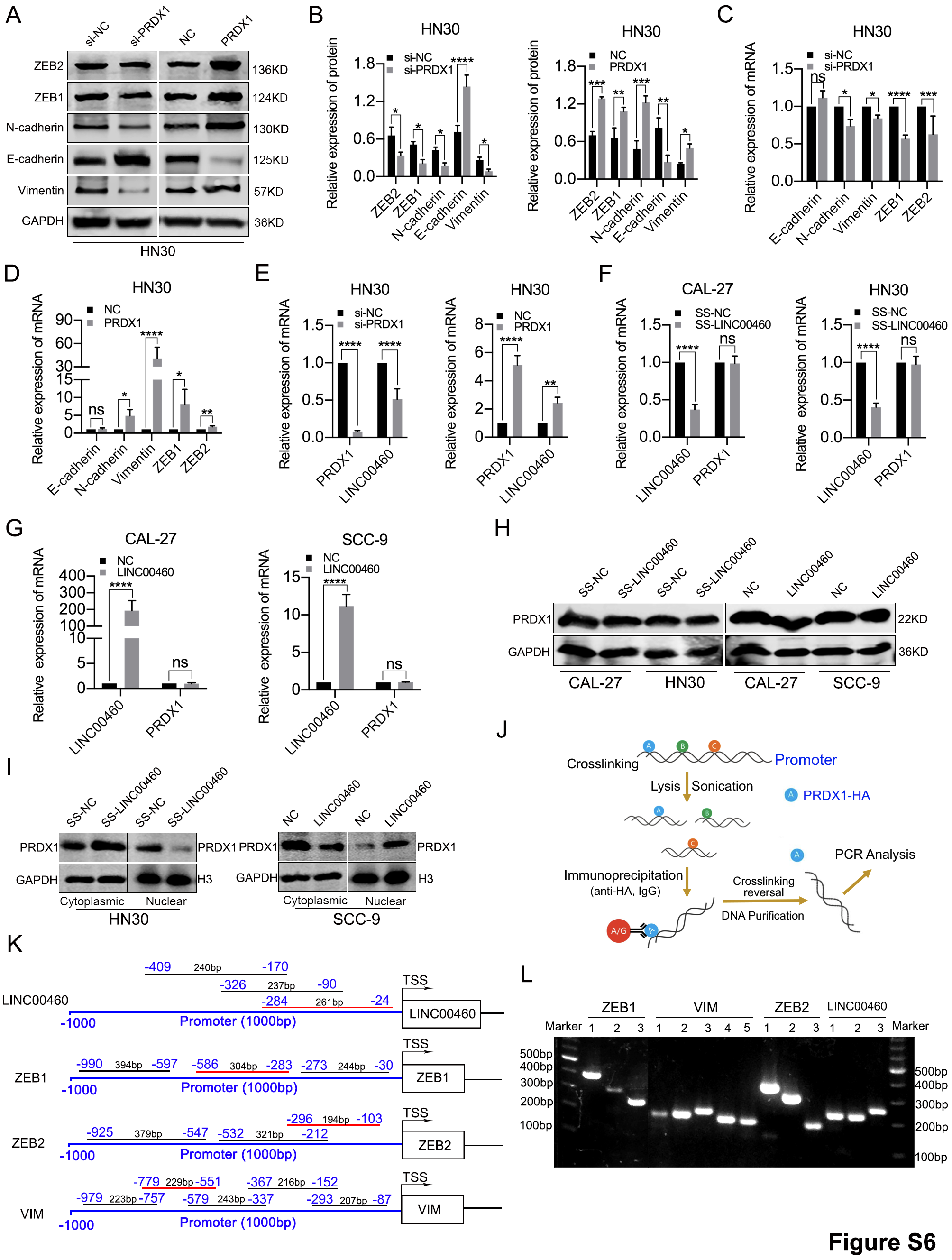

Supplement: Supplementary file 11 — Figure S6. PRDX1 affected EMT in HNSCC cells and promoted the transcription of LINC00460 and EMT-related genes. (A, B) The expression of EMT markers (E-cadherin, N-cadherin, Vimentin, ZEB1 and ZEB2) was detected by Western blot analysis when PRDX1 was knocked down or overexpressed in HN30 cells. (C, D) The expression of EMT-related genes (E-cadherin, N-cadherin, Vimentin, ZEB1 and ZEB2) was detected by qRT-PCR when PRDX1 was knocked down (C) or overexpressed (D) in HN30 cells. (E) qRT-PCR analysis of LINC00460 expression in HN30 cells when PRDX1 was knocked down or overexpressed. (F, G) qRT-PCR analysis of PRDX1 expression when LINC00460 was knocked down (F) or overexpressed (G) in HNSCC cells. (H) The protein level of PRDX1 was analyzed by Western blotting when LINC00460 was knocked down or overexpressed in HNSCC cells. (I) The protein levels of PRDX1 in nuclear and cytoplasmic fractions were analyzed by Western blotting in HN30 cells transfected with SS-LINC00460 and in SCC-9 cells transduced with LINC00460. (J) The process of the ChIP assay performed in our study. (K) The specific primers for the promoter region were designed for ChIP assays. TSS, Transcription Start Site. (L) The ChIP primers provided specific amplification. *p < 0.05, **p < 0.01, ***p < 0.001, ****p < 0.0001, ns: no significance. (TIF 1158 kb) [file 13046_2019_1364_MOESM11_ESM.tif]

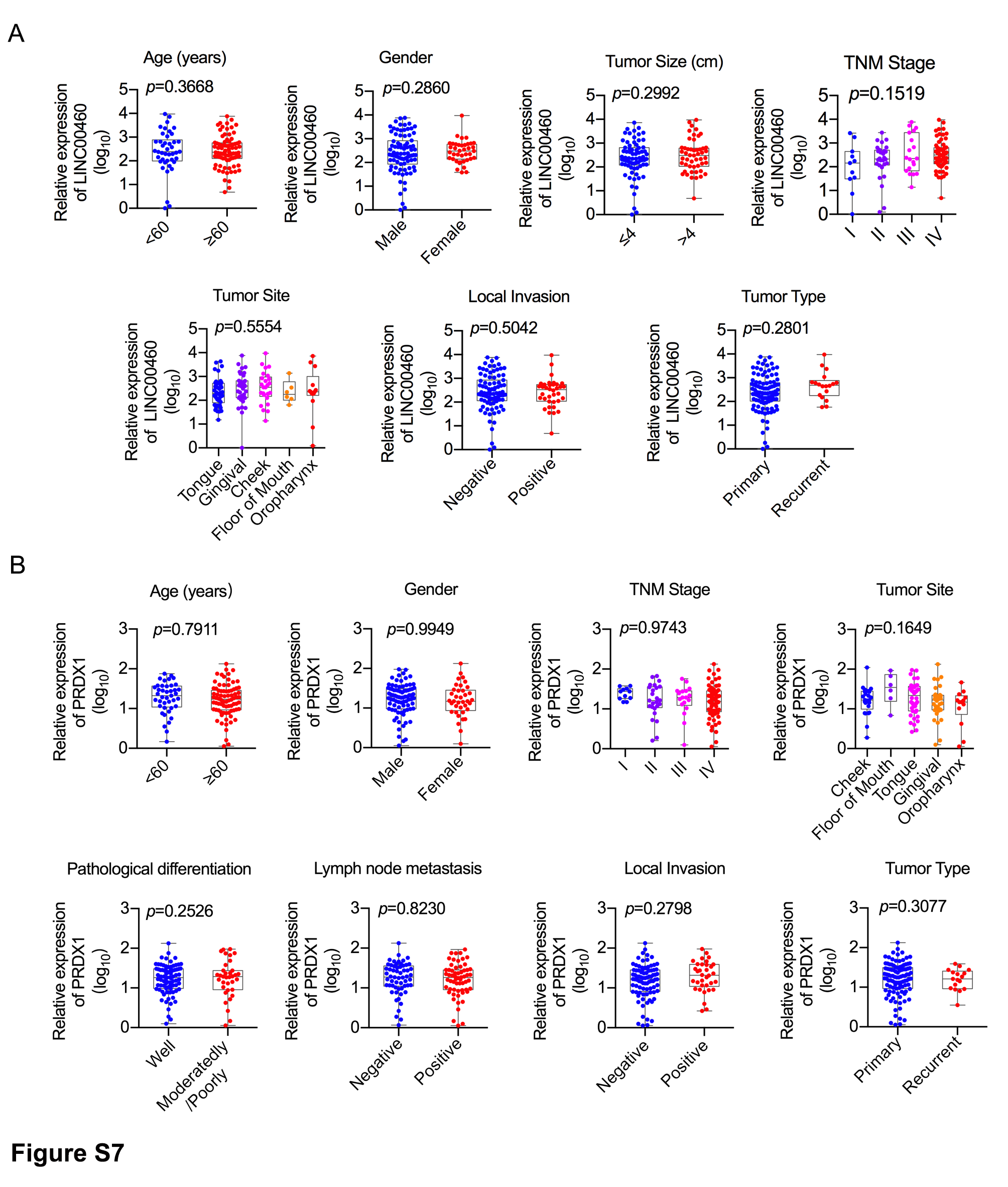

Supplement: Supplementary file 12 — Figure S7. Analysis of the association between LINC00460 and PRDX1 expression and clinical significance in HNSCC tissues. (A) The associations between the relative expression of LINC00460 and clinical parameters were investigated in patients with HNSCC (p > 0.05). (B) The associations between the relative expression of PRDX1 and clinical parameters were investigated in patients with HNSCC (p > 0.05). (TIF 1013 kb) [file 13046_2019_1364_MOESM12_ESM.tif]
